# Genomic Diversity of Bacteriophages Infecting the Genus *Acinetobacter*

**DOI:** 10.3390/v14020181

**Published:** 2022-01-19

**Authors:** Hugo Oliveira, Rita Domingues, Benjamin Evans, J. Mark Sutton, Evelien M. Adriaenssens, Dann Turner

**Affiliations:** 1 Centre of Biological Engineering, University of Minho, Campus de Gualtar Braga, 4710-057 Braga, Portugal; hugooliveira@deb.uminho.pt (H.O.); ritadomingues1898@gmail.com (R.D.); 2 Norwich Medical School, University of East Anglia, Norwich NR4 7TJ, UK; benjamin.evans@uea.ac.uk; 3 United Kingdom Health Security Agency, Research and Evaluation, Porton Down, Salisbury SP4 OJG, UK; mark.sutton@phe.gov.uk; 4 Quadram Institute Bioscience, Norwich Research Park, Norwich NR4 7UQ, UK; evelien.adriaenssens@quadram.ac.uk; 5 Department of Applied Sciences, Faculty of Health and Applied Sciences, University of the West of England, Bristol BS16 1QY, UK

**Keywords:** *Acinetobacter*, bacteriophages, comparative genomics, phage therapy, bioinformatics

## Abstract

The number of sequenced *Acinetobacter* phage genomes in the International Nucleotide Sequence Database Collaboration has increased significantly in recent years, from 37 in 2017 to a total of 139 as of January 2021 with genome sizes ranging from 31 to 378 kb. Here, we explored the genetic diversity of the *Acinetobacter* phages using comparative genomics approaches that included assessment of nucleotide similarity, shared gene content, single gene phylogeny, and the network-based classification tool vConTACT2. Phages infecting *Acinetobacter sp.* are genetically diverse and can be grouped into 8 clusters (subfamilies) and 46 sub-clusters (genera), of which 8 represent genomic singletons (additional genera). We propose the creation of five new subfamilies and suggest a reorganisation of the genus *Obolenskvirus*. These results provide an updated view of the viruses infecting *Acinetobacter* species, providing insights into their diversity.

## 1. Introduction

Advances in sequencing technologies and bioinformatics tools have enabled in-depth exploration and revealed the vastness of bacteriophage (phage) biodiversity. The diversity of phages is exemplified by the number of novel genes, little nucleotide sequence similarities between many sequenced phages, and, increasingly, by the sheer number of distinct viral sequences being uncovered through metaviromics of various environmental niches [1].

Comparative genomics has emerged as a fundamental tool for the investigation of phage genomes, allowing the identification of related phage genomes and providing an opportunity to identify and evaluate relationships both within and between clades [2]. Genome-based metrics are now used by the Bacterial Viruses Subcommittee of the International Committee on the Taxonomy of Viruses (ICTV) for the delineation and assignment of formal taxonomic ranks [3]. Comparisons at the nucleotide sequence and whole proteome levels have shown that, for lytic phages at least, evolution of these viruses appears to be predominantly vertical and that some clades of phages represent ancient lineages (e.g., the N4-like phages of the family *Schitoviridae*), given that closely related phages have been isolated from disparate geographic locations, environments, and times.

*Acinetobacter baumannii* is an opportunistic pathogen which is associated with a range of nosocomial infections. Due to an ability to persist for long periods in the environment and to develop resistance to multiple antibiotic classes, including broad spectrum beta-lactams such as third generation cephalosporins and carbapenems, fluoroquinolones, aminoglycosides, tigecycline, and colistin, extensively drug-resistant and even pan-drug resistant strains are emerging in clinical settings worldwide [4,5]. Infections caused by *A. baumannii* are more common in intensive care units and surgical wards, manifesting as ventilator-associated pneumonia or bacteraemia but also as soft-tissue injury and urinary tract and gastrointestinal infections [6,7].

Members of the genus *Acinetobacter* are defined phenotypically as Gram negative, catalase positive, oxidase negative, non-fermenting coccobacilli. There are currently 69 species recognised within the genus that are validly published and possess a correct name (https://lpsn.dsmz.de/genus/Acinetobacter, accessed on 21 June 2021). *A. baumannii* is part of the *Acinetobacter calcoaceticus-baumannii* (Acb) complex, which includes species with similar phenotypic and genotypic properties. The Acb complex is comprised of five pathogenic species: *A. baumannii*, *Acinetobacter pittii*, *Acinetobacter nosocomialis*, *Acinetobacter seifertii,* and *Acinetobacter dijkshoorniae,* as well as the non-pathogenic species *Acinetobacter* *calcoaceticus* [8].

Although strains of *A. baumannii* show limited diversity among core genes, the genome is both plastic and dynamic, which is shown by significant variance in accessory gene content. A significant factor in the accessory genome variance is the acquisition of a wide variety of mobile genetic elements as well as hotspots of recombination, which has contributed to the evolution of extensive and pan-drug resistant phenotypes [9]. Notably, comparative analysis of isolates from international clonal lineage I has shown hotspots of recombination associated with capsular polysaccharide, lipooligosaccharide, and outer membrane proteins—surface structures which are commonly targeted by phages for adsorption [10,11].

The rapid accumulation of resistance genes has severely depleted the number of antibiotics available as effective treatment options for infections caused by the Acb complex. Increasingly, resistance to carbapenems and polymixins threatens to further compound this problem [12]. Collectively, this has resulted in the designation of *A. baumannii* as a critical target for the development of new therapeutic options by the World Health Organisation (WHO) (https://www.who.int/medicines/publications/WHO-PPL-Short_Summary_25Feb-ET_NM_WHO.pdf, accessed on 21 June 2021). With the lack of new antibiotics in development, the use of phages (phage therapy) or phage-derived proteins as a therapeutic option, often applied as a combinatorial therapy with existing classes of antibiotics, is gaining increased attention. As importantly, the investigation and analysis of phage genomes and gene products can lead to the development of novel molecular tools to study this important pathogen.

In 2017, there were 37 *Acinetobacter* phage genomes available in public databases. Based on the number of shared proteins, nucleotide identity, and genomic architecture, the *Acinetobacter* viral population was comprised of six discrete clusters and two singletons [13]. Notably, half of the protein-coding genes had no assigned functions, illustrating the vast amount of the so-called “genetic dark matter” in these viral genomes. Since then, the number of available *Acinetobacter* phage genomes has increased substantially, necessitating a re-evaluation of these data. Here, we present an updated analysis of all sequenced *Acinetobacter* phages held in the International Nucleotide Sequence Database Collaboration (INSDC) as of the 21st of January 2021. The *Acinetobacter* phage genomes were re-annotated and examined using a comparative bioinformatics approach based upon whole genome alignments, protein clustering, and phylogenetic analysis. We highlight the taxonomic population structure of these phages and identify core, accessory, and unique protein-coding genes present within, and shared between, clades.

## 2. Materials and Methods

### 2.1. Genome Annotation

*Acinetobacter* phage genomes in the FASTA file format were downloaded from GenBank (21 January 2021). Genome sequences were compared to each other using BLASTN and edited so that they were colinear by taking the reverse complement and/or reopening the sequence. To ensure consistency in gene calls for downstream annotation and analyses, all genomes were re-annotated using Prokka [14] using a custom ‘*Caudovirales’* database (http://millardlab.org/bioinformatics/lab_server/phage-genome-annotation/, accessed on 21 January 2021). For each annotated genome, translated ORF sequences were searched against hidden Markov models (HMM) profiles downloaded from the VOG database (release number vog204) [15] using hmmscan with an e-value cut-off of 1 × 10^−5^. Matches to pVOG profiles were considered significant at an e-value of ≤1 × 10^−5^ with ≥35% coverage of the profile HMM.

### 2.2. Global Profiling of Acinetobacter Phage Genomes

To provide a snapshot of the relationship between the *Acinetobacter* phages and the wider phage population, a gene-sharing network of all phage genomes held in GenBank (24 January 2021) was constructed using the INPHARED pipeline [16] and vConTACT2 0.9.19, using DIAMOND v2.0.7.145 was applied for protein clustering and ClusterOne v1.0 for protein and viral cluster analyses. The resulting network was visualised using Cytoscape (version 3.8.2) [17] and annotated with Adobe Illustrator (Adobe Inc., San Jose, CA, USA).

### 2.3. Genome Clustering

The diversity of *Acinetobacter* phage genomes was assessed using DNA sequence comparisons, protein clustering, and single-gene phylogenies. At the DNA level, VIRIDIC was used for an all-versus-all nucleotide sequence comparison of the genomes with the following parameters: species threshold 95, genus threshold 65, -word_size 7, -reward 2, -penalty -3, -gapopen 5, and -gapextend 2 [18].

For the identification of orthologous groups of proteins, two approaches were used. First, translated ORF sequences were clustered into groups using PIRATE [19] by using parameters of 30–40% minimum sequence identity and ≥50% coverage of high-scoring pairs. Amino acid sequences for each group of proteins were extracted and further functional inferences obtained using InterProScan version 5.48–83.0 [20]. Sequences comprising of protein groups were first aligned using Clustal Omega version 1.2.4 [21] and then used to search the UniRef30_2020_06 database using HHblits with a single iteration and an e-value threshold of 1 × 10^−5^. Alignments produced by HHblits were converted to hmm profiles using the tool hhmake and then queried against the pdb70_from_the mmcif_210127 database using HHsearch. Remote homologies identified using HHsearch were used to further refine the phage genome annotations where the results were considered significant if the probability was greater than 75% and the e-value was <1 × 10^−3^.

As a secondary measure, *Acinetobacter* phage genomes were also analysed with Phamerator to sort genes into phamilies of related sequences (phams if shared by at least two proteins; orphams for unique proteins) using kclust alignments [22]. A structured query language (SQL) database containing the phage genome GenBank files was created and imported to Phamerator using a Virtual Machine (VM Virtual Box, Oracle Corporation, Austin, TX, USA). Next, SplitsTree (version 5_2_28-beta) [23] was used to plot the genome diversity based on their shared protein content, which was obtained by Phamerator.

The maximum likelihood phylogenetic trees were reconstructed using IQ-Tree version 1.6.12 [24] from alignments generated using Clustal Omega, and implemented the fast method of the substitution model selection with ModelFinder, SH-aLRT test and ultrafast bootstrap approximation (UFBoot) with 1000 replicates [25,26,27]. The phylogenetic trees were visualised using ITOL [28]. The comparative genome diagrams were prepared using Clinker [29], using a BLASTp identity threshold of 30% and edited in Adobe Illustrator (Adobe Inc., San Jose, CA, USA).

### 2.4. Cluster Assignment

Here, we employ the terms cluster, to represent the subfamily-level relationships, and sub-cluster, to denote genera using the guidelines of the Bacterial Viruses Subcommittee of the International Committee on the Taxonomy of Viruses (ICTV). Phages were grouped into clusters (subfamilies) based on a minimum of 30% shared proteins identified using PIRATE and assigned a letter to represent each individual cluster. These clusters were further divided into sub-clusters (genera) using a threshold of ≥65% nucleotide sequence similarity and assigned a number—for example, cluster A, sub-cluster 1 (A1). Below these thresholds, genomes identified as being unrelated to other *Acinetobacter* phages or to the wider population of sequenced phages were assigned as genomic singletons (unique genera).

### 2.5. Transmission Electron Microscopy

Phages RPH5R, RPH2R, TRS2, and TRS1 were isolated from wastewater treatment plants. Virions were pelleted from PEG-precipitated or CsCl purified samples by centrifugation at 25,000× *g* for 65 min. Pellets were resuspended in 100 µL of 0.1 M ammonium acetate (pH7.2). Specimens for transmission electron microscopy were prepared by the addition of 5 µL to 400 mesh carbon-coated formvar grids and stained by the addition of an equal volume of 2% *w/v* uranyl acetate. Grids were examined using a Phillips CM10 equipped with a Gatan Orius SC1000 CCD camera (Gatan, Pleasanton, CA, USA).

## 3. Results

### 3.1. Taxonomy of the Acinetobacter Phages

A dataset of 134 *Acinetobacter* phage genomes was prepared (Table S1) after the exclusion of four phages, PBAB08 [MG366114], PBAB25 [MG366115], φFG02 [MT648818], and φC001 [MT648819], due to the presence of multiple frameshift assembly errors. The single representative of the class *Leviviricetes*, species *Appevirus quebecense*, AP205 was also excluded.

To assess the relationships among the *Acinetobacter* phages at the nucleotide sequence level, intergenomic similarities were calculated reciprocally between pairs of viral genomes using VIRIDIC [18]. Using the ICTV recommended criteria for the demarcation of genera [30], we identified a total of 46 sub-clusters based on a threshold of a ≥65% nucleotide sequence similarity (Figure S1). The *Acinetobacter* phages can therefore be classified into 46 genera, of which 14 correspond to the ratified ICTV taxa: *Zedzedvirus* (A1), *Lazarusvirus* (A2), *Hadassahvirus* (A3), *Lasallevirus* (A4), *Acajnonavirus* (A5), *Obolenskvirus* (B4), *Saclayvirus* (C3), *Friunavirus* (E2), *Pettyvirus* (E3), *Daemvirus* (E4), *Vieuvirus* (F3), *Lokivirus* (G1), *Presleyvirus* (H1), *Exceevirus* (H2), and *Metrivirus* (singleton ME3).

Several of the individual sub-clusters exhibit a nucleotide sequence similarity of >30%, but below the recommended genus threshold of 65–70%, suggesting that subfamily level relationships may exist between them. While nucleotide sequence similarity provides a robust measure for closely related phages, the examination of shared protein content provides a measure of the intra-cluster variability, identification of signature genes, and reveals more distant relationships at the subfamily level or higher. To assess these relationships, a pan-genome analysis of the 15,734 proteins encoded by the *Acinetobacter* phages was performed using PIRATE and Phamerator. Using PIRATE, proteins were clustered using the criteria of a >30% sequence identity and a >50% query coverage. A total of 1492 groups, consisting of two or more proteins and 2444 unique proteins, were identified using this approach (File S1). Phamerator identified 1650 phams of two or more proteins and 2825 orphams (64%) composed of unique sequences. We define (i) core proteins as those that are present in all members of a cluster or sub-cluster; (ii) accessory proteins as those present in two or more, but not all of the members; and (iii) unique proteins as those found only in a single phage genome.

**Figure 1:**
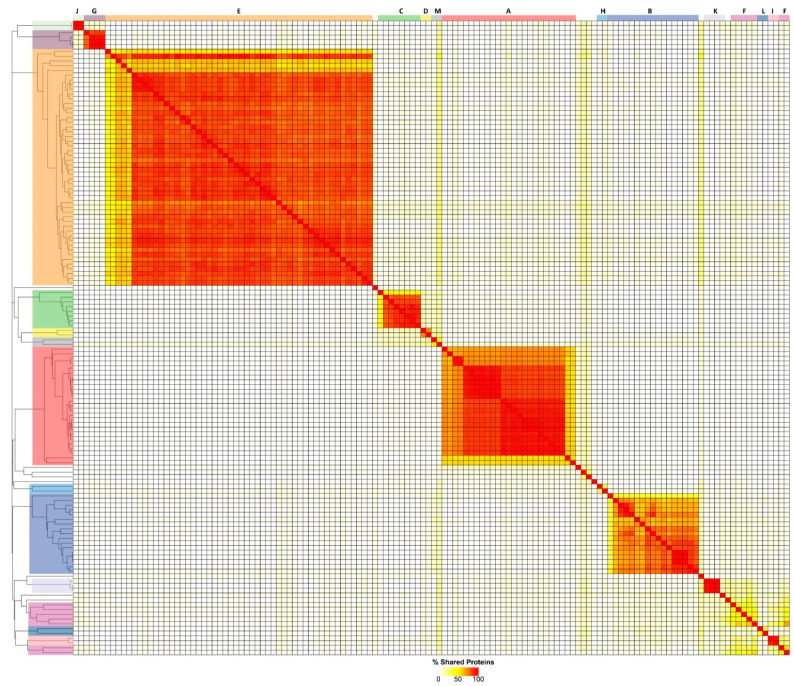
Heatmap of the percentage of proteins shared between pairs of *Acinetobacter* phage genomes. The heatmap was produced from the output of the PIRATE pan-genome analysis and hierarchically clustered using the complete linkage method in R. Clusters are indicated by coloured blocks at the top of the heatmap and within the dendrogram according to the key: 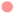 A; 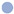 B; 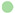 C; 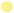 D; 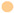 E; 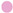 F; 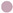 G; 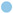 H; 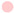 I; 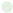 J; 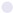 K; 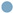 L; 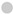 M.

Hierarchical clustering of the shared protein content obtained from the Phamerator and PIRATE pan-genome analyses demonstrated that 33 of the sub-clusters could be grouped to form a total of 8 clusters (A, B, C, E, F, H, L, and M) representing subfamily level relationships that exhibited, at minimum, 11 conserved core genes (Figure 1 and Table 1). At present, only two of these clusters are recognised as formal ICTV taxa, the subfamilies *Twarogvirinae* (Cluster A) and *Beijerinckvirinae* (Cluster E). While no wider relationships within the *Acinetobacter* phages were identified for five of the sub-clusters (D1, G1, I1, J1, and K1), it is possible that they may be assigned subfamily status with the isolation of related phages in the future. The remaining eight phages represented single species that exhibited no significant similarity in nucleotide or shared protein content and are assigned as genomic singletons.

**Figure 2:**
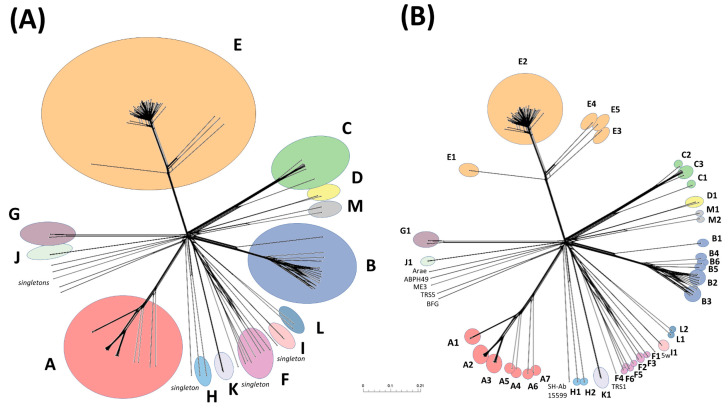
Diversity of *Acinetobacter* phage genomes. From a total of 15,734 predicted genes of the 134 *Acinetobacter* phage genomes, it generated 1650 phams of two or more proteins and 2825 orphams (64%) composed of unique sequences were identified in Phamerator. The resulting network was to visualize the genomics diversity with SplitsTree using “genome content distance” mode. Phages were assigned to (**A**) clusters and (**B**) subclusters if sharing >35 genes or lower, respectively. The scale bar indicates 0.01 substitution per site (number of gene differences, presence or absence, per gene site). Clusters and subclusters are marked by ellipses and bold letters according to the key: 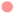 A; 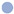 B; 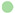 C; 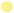 D; 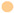 E; 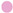 F; 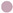 G; 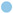 H; 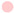 I; 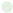 J; 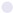 K; 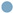 L; 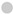 M. Singleton phages Arae, ABP49, ME3, TRS5, BFG and SH-Ab 15599 are named in (**B**).

The relationships between the *Acinetobacter* phages were illustrated by a network phylogeny created using the entire complement of proteins, which was converted to a binary matrix to denote the presence or absence of each protein within each individual phage genome. The network reflected the assignment of sub-clusters from the nucleotide sequence comparisons and revealed the more distant subfamily level relationships between some clusters, which are represented by a deeper branching structure (Figure 2).

**Figure 3:**
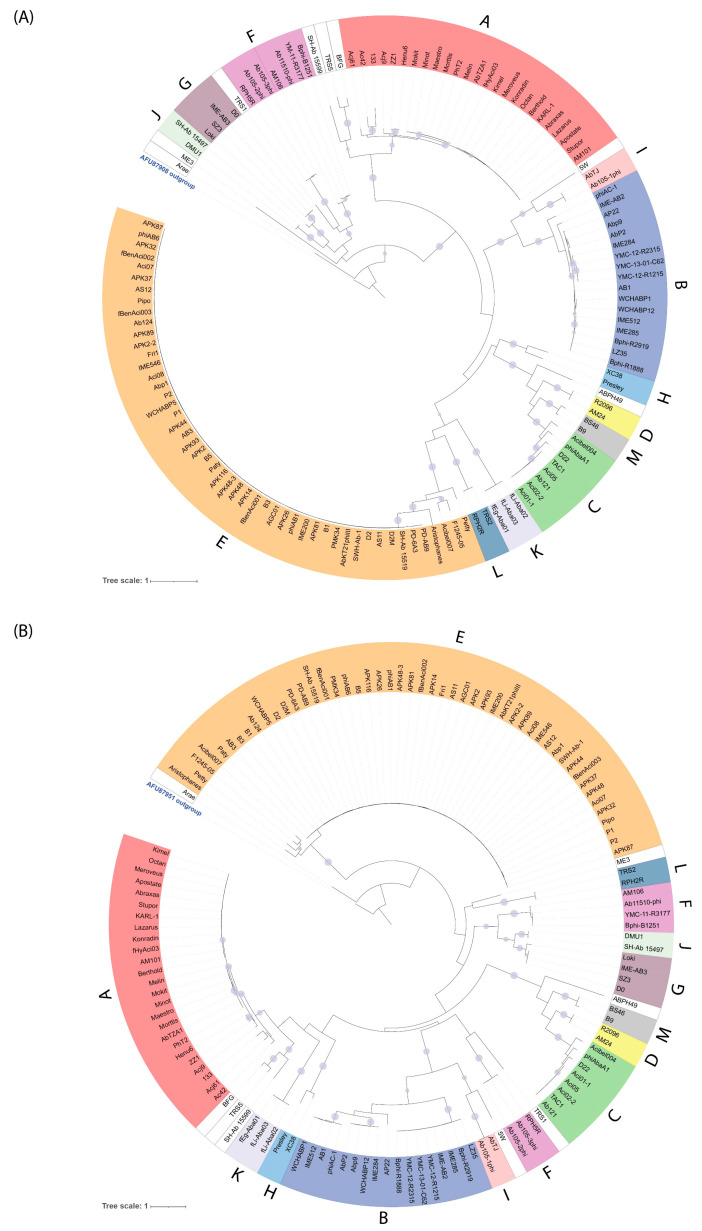
Phylogenetic analysis of (**A**) the portal vertex and (**B**) major capsid proteins of the *Acinetobacter* phages. The maximum likelihood phylogenetic trees were inferred from alignments generated by Clustal Omega with VT + F + R3 (portal) and Q.pfam + F + G4 (capsid) models of evolution and 1000 bootstrap replicates and SH-Alrt test using IQ-TREE and visualised using ITOL. The trees were rooted using the portal or major capsid protein from *Caulobacter* phage CcrColossus, indicated by bold blue type. Ultrafast bootstrap values (≥95%) are marked with filled circles, with the size proportional to the bootstrap value. Clusters are denoted by coloured arcs and labels on the outer edge of the tree (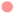 A; 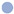 B; 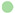 C; 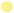 D; 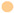 E; 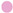 F; 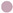 G; 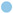 H; 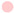 I; 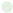 J; 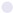 K; 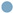 L; 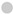 M). The scale bar represents substitutions per site.

With the exception of cluster F, which is discussed below, the assignment of subfamily level clusters was also supported by a low standard deviation of the average genome size, % G + C content, and the number of protein coding genes, as well as by phylogenetic analysis of the major capsid and portal vertex proteins (Figure 3).

**Figure 4:**
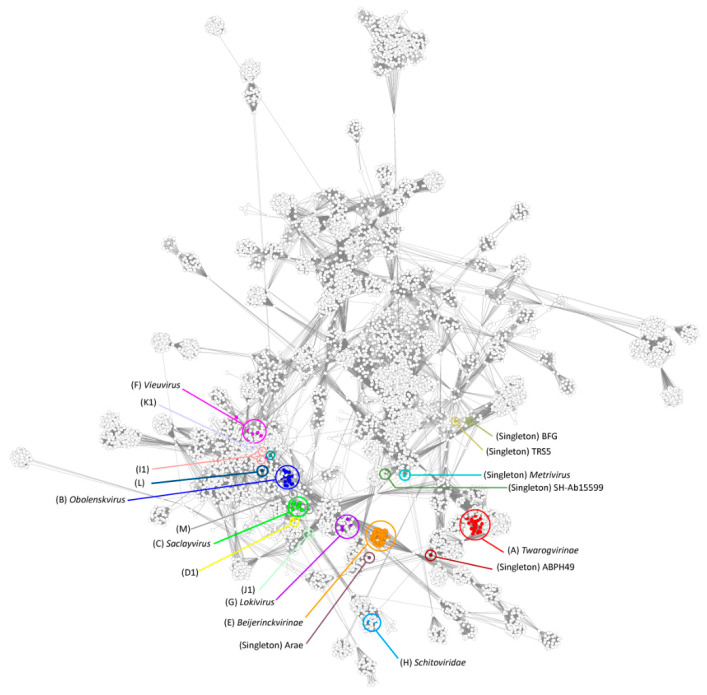
vConTACT2 network analysis. The network represents a subset of dsDNA prokaryotic virus genomes. Each circle (node) represents a genome and connecting lines (edges) represent the similarity between genomes based on shared clusters of proteins. *Acinetobacter* phage genomes are highlighted as filled coloured nodes with cluster designations provided in parentheses, and ICTV-ratified genus and subfamily names are presented in italics. Clusters are coloured according to the key: 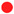 A; 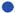 B; 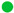 C; 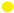 D1; 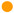 E; 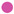 F; 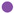 G; 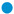 H; 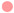 I; 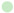 J1; 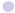 K; 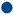 L; 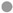 M; Singleton phages: 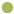 BFG; 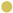 TRS5; 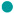 5W; 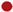 ABPH49; 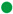 TRS1; 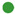 SH-Ab 15599; 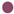 Arae; 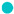 ME3.

vContact2 was used to assess the relationship of the *Acinetobacter* phages with the wider phage population (Figure 4). In the resulting network, the *Acinetobacter* phages fell into groups that reflect those defined by their nucleotide similarity and shared protein content, but more distant relationships were also revealed. Members of the genus *Lokivirus* (sub-cluster G1) formed part of a larger clade of siphoviruses that include members of the genera *Septimatrevirus, Kilunavirus,* and *Titanvirus*. Singleton phages SH-Ab 15599 and ABPH49 clustered with members of the *Ackermannviridae* and *Vequintavirinae*, respectively. The sub-clusters representing the temperate *Acinetobacter* phages fell within a much larger grouping that mainly consisted of phages capable of lysogeny.

The number of sequenced *Acinetobacter* phages has increased substantially since the last comparative analysis of phages infecting this bacterial genus, revealing a diverse and heterogeneous population [13]. While existing phage genera have expanded to include new members, many distinct new species representatives have been isolated. In addition to the ICTV-ratified subfamilies *Twarogvirinae* and *Beijerinckvirinae*, we propose the creation of five new subfamilies, and suggest a reorganisation of the genus *Obolenskvirus* by splitting this genus and creating a new subfamily to encompass the new genera. Herein, short descriptions of each of the major clusters (subfamilies) and their constituent sub-clusters (genera) are provided. We also focus on three themes: host adsorption, nucleotide modifications, and the lysis systems of these diverse groups of phages.

### 3.2. T4-like Phages Infecting Acinetobacter sp.

Cluster A is comprised of 25 T4-like phages that that can be grouped into seven sub-clusters (A1–A7) that infect the host species *A. baumannii*, *A. johnsonnnii,* and *A. pittii*. The taxonomy of the T4-like phages has recently undergone a significant re-evaluation, resulting in the creation of two families, *Straboviridae* and *Kyanoviridae* (ICTV taxonomic proposal 2021.082B.A.v1). All of the T4-like *Acinetobacter* phages are classified within the subfamily *Twarogvirinae* of the *Straboviridae*. These phages exhibit a typical T4-like virion morphology, which comprises of a prolate head and a contractile tail that terminates in a baseplate with long and short tail fibers. At minimum, these phages exhibit 22% nucleotide sequence similarity but share a total of 107 core and 255 accessory protein groups, of which 81 and 225, respectively, are unique to the *Twarogvirinae* and are not found in any other cluster of *Acinetobacter* phages.

### 3.3. Phages of the Obolenskvirus Represent a New Subfamily of Myoviruses

**Figure 5:**
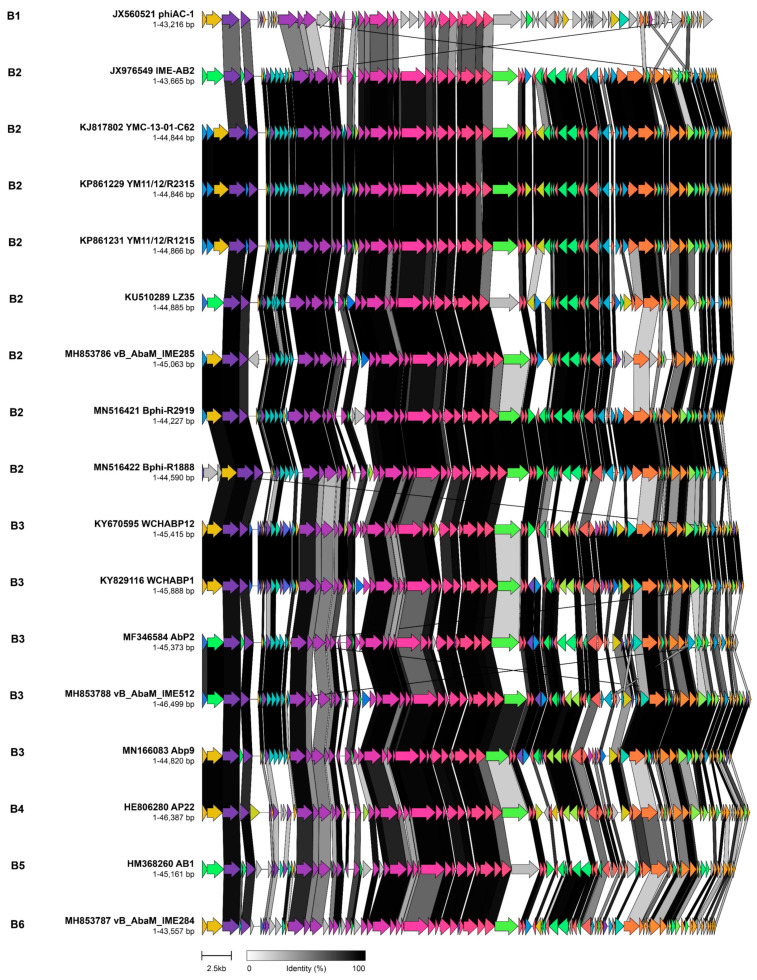
Comparative genome alignment of phages comprising sub-cluster B. Phage genomes are presented alongside their designated sub-cluster, accession number, name and genome length. Coding sequences are represented by arrows, coloured to reflect homologous groups identified by Clinker, and are linked by grey bars shaded to represent the percentage amino acid identity, as indicated in the legend.

Cluster B has six sub-clusters (genera B1–B6). It contains *Acinetobacter* phages currently classified within the genus *Obolenskvirus* and a single outlier, phiAC-1, that shares an average of 38.5% of its coding sequences and 25–31% sequence similarity with members of this cluster. Here we propose that the 17 cluster B phages represent a discrete subfamily consisting of 15 phage species. The genomes exhibit a common modular and syntenic organization with a total of 21 core proteins present across the subfamily, predominantly representing the virion structural and assembly proteins (Figure 5).

One interesting feature is that, in all members of this cluster, the virion structure and assembly module is separated between the head morphogenesis protein and prohead protease by several ORFs of unknown function that exhibit the differences in composition between the member species. While there is a high degree of shared protein content (55.8–93.6% of ORFs) between each of the 17 phages in Cluster B, we noted that the nucleotide similarity falls below the recommended ICTV threshold of 65–70%. By this criterion, the *Obolenskvirus* could be split into six separate genera.

### 3.4. The Cluster Comprising Saclayvirus, Acibel004, and phiAbaA1 Represent a New Subfamily of Myoviruses

**Figure 6:**
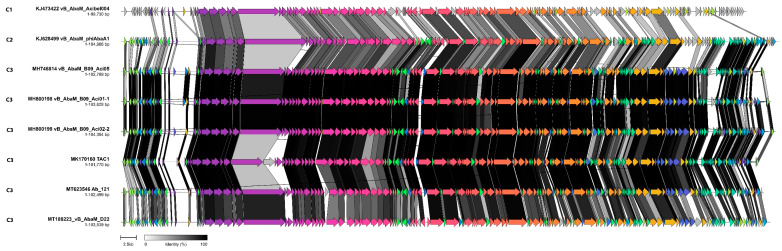
Comparative genome alignment of *Acinetobacter* phages comprising cluster C. Phage genomes are presented alongside their designated sub-cluster, accession number, name and genome length. Coding sequences are represented by arrows, coloured to reflect homologous groups identified by Clinker, and are linked by grey bars shaded to represent the percentage amino acid identity, as indicated in the legend.

Cluster C is comprised of eight myoviruses (Acibel004, phiAbaA1, D22, TAC1, B09_Aci01-1, B09_Aci02-2, B09_Aci05, and Ab121) separated into three sub-clusters (genera C1–C3). These phages possess genomes ranging from 99.7 to 104.4 kb with 151 to 173 protein coding genes in a syntenic and modular arrangement (Figure 6).

Transmission electron micrographs of Acibel004 and B09_Aci05 show a common morphotype with a characteristic distal tail structure appearing to show a triangular cluster of tail fibers that converge at the apex [31,32]. At the nucleotide sequence level, Acibel004 (sub-cluster C1) and phiAbaA1 (sub-cluster C2) can be classed as outliers, exhibiting a ~30% and 60% sequence similarity and an average similarity of 49.5 and 80.5% in their proteins, respectively, when compared to other members of cluster C. The remaining phages, TAC1, D22, B09_Aci01-1, B09_Aci02-2, B09_Aci05, and Ab121 (sub-cluster C3) formed a monophyletic clade characterised by an 83–94% sequence similarity and 129 core proteins shared. Three of these phages, B09_Aci05, B09_Aci01-1, and B09_Aci02-2 are currently classified within the genus *Saclayvirus* (ICTV Taxonomic Proposal 2019.072B). Given that there are 69 core protein groups shared across these eight phages, we propose the creation of a new subfamily tentatively named as the “Astridvirinae”, after Queen Astrid Military Hospital where Acibel004 was first isolated.

### 3.5. AM24 and R2096 Represent a New Genus of Myoviruses

**Figure 7:**
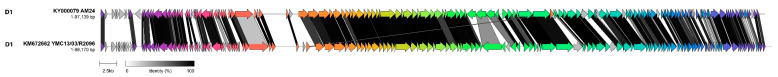
Comparative genome alignment of phages comprising sub-cluster D1. Phage genomes are presented alongside their designated sub-cluster, accession number, name and genome length. Coding sequences are represented by arrows, coloured to reflect homologous groups identified by Clinker, and are linked by grey bars shaded to represent the percentage amino acid identity, as indicated in the legend.

Phages YMC13/03/R2096 (R2096) and AM24 remain the only two members of cluster D1. This grouping was supported by a 78.8% nucleotide sequence similarity and 70.2% shared proteins (Figure 7). Of the 115 core proteins, 87 were exclusive to this cluster, and these two species differ in gene content within the early and genome replication modules. Both phages have a myovirus morphology, identical genome organisation, and form plaques with opaque halos, which are indicative of depolymerase activity. AM24 has been shown to exhibit specificity for the K9 capsule type [33,34].

### 3.6. Expansion of the Subfamily Beijerinckvirinae

Cluster E represented the largest group of phages infecting *Acinetobacter* sp., comprising five sub-clusters (genera E1–E5). They are members of the *Autographiviridae* subfamily *Beijerinckvirinae* that has now expanded to include a total of 50 isolates. They are podoviruses that possess small genomes (c. 40 kb) with short direct terminal repeats and encode a DNA-dependent RNA polymerase, which is characteristic of the family *Autographiviridae*. Sub-cluster E2, delineated as the genus *Friunavirus*, represents the largest and most conserved genus of *Acinetobacter* phages, comprising of 47 highly similar genomes sharing a >70% sequence similarity and >70% shared proteins. These phages differ primarily at the middle and C-terminal regions of the tailspike protein responsible for polysaccharide depolymerase activities, in the complement of some putative early genes, and the presence or absence of homing endonucleases. We noted that two phages, Pipo [MW366783] and Paty [MW366784], while described as *Klebsiella* phages in GenBank, are more likely to be *Acinetobacter* phages given the degree of nucleotide similarity and shared protein content with constituent members of the *Friunavirus*. A further four isolates, Acibel007 (sub-cluster E4), Aristophanes (sub-cluster E1), F1254-05 (sub-cluster E5), and Petty (sub-cluster E3), represent new species in individual genera within the subfamily, exhibiting a ~25% sequence similarity to members of the *Friunavirus* and sharing 21 core proteins when the partial genome of AB3 is excluded.

### 3.7. Temperate Acinetobacter Phages Encode Diverse Complements of Accessory Proteins

The number of isolated or induced temperate *Acinetobacter* phages has expanded in recent years. Previously, cluster F was comprised of two phages, Bphi-B1251 and YMC11/11/R3177 (R3177), which we now define as sub-cluster F3 and represents the members of the genus *Vieuvirus*. Relative to Bphi-B1251, R3177 exhibits a 62.4% sequence similarity and has 56.4% shared proteins, where the difference is accounted for by the localised differences in the gene content, which suggests these two related phages might undertake different lifestyles [13]. A further five siphoviruses, AM106, Ab11510-phi RPH5R, Ab105-2phi, and Ab105-3phi (sub-clusters F1, F2, F4, F5 and F6, respectively), exhibit a degree of sequence similarity and shared protein content with Bphi-1251 and R3177. These phages share a significant number of virion structural and genome replication genes and differ primarily in the complement of small accessory and unique proteins. On this basis, we have tentatively grouped these predominantly temperate *Acinetobacter* phages into a single cluster. However, we note that this assignment is not supported by the phylogenetic analysis of the portal and major capsid proteins. Further work is required to induce or isolate new relatives of these species before their taxonomy can be resolved in more detail.

**Figure 8:**
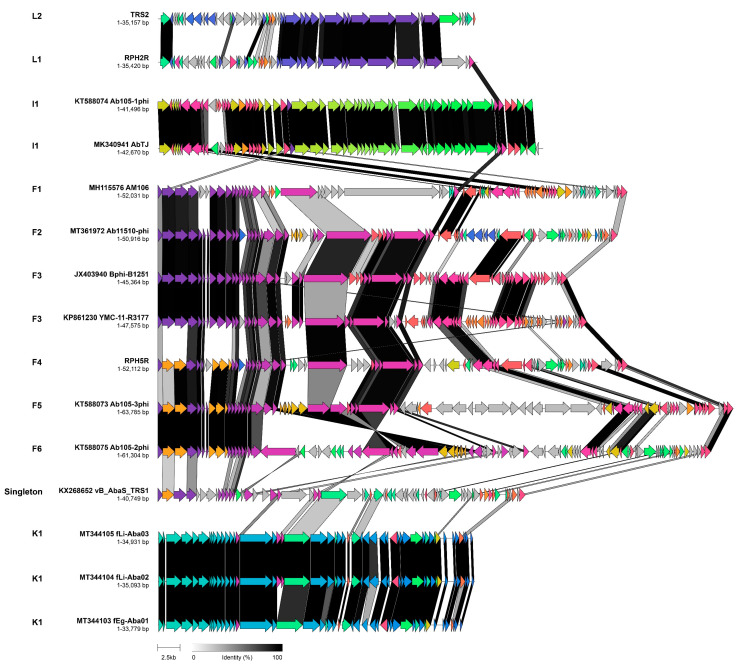
Comparative genome alignment of sub-clusters comprising of temperate phages infecting *Acinetobacter sp*. Phage genomes are presented alongside their designated sub-cluster, accession number, name and genome length. Coding sequences are represented by arrows, coloured to reflect homologous groups identified by Clinker, and are linked by grey bars shaded to represent the percentage amino acid identity, as indicated in the legend.

**Figure 9:**
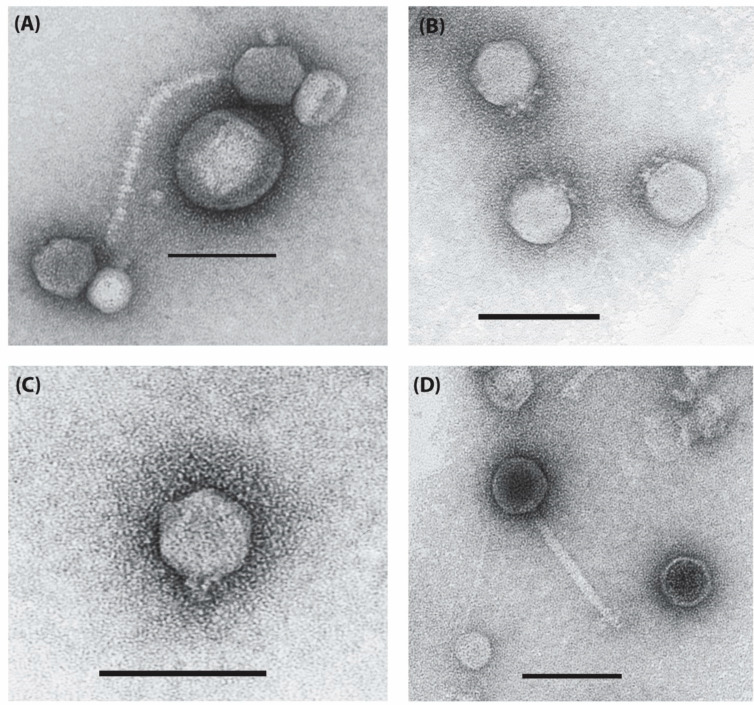
Novel temperate phages of *A. baumannii*. Transmission electron micrographs of (**A**) RPH5R; (**B**) RPH2R (**C**) TRS2; and (**D**) TRS1, negatively stained with 2% *w/v* uranyl acetate. Scale bars represent 100 nm.

Interestingly, all of the temperate *Acinetobacter* phages, whether induced from the host lysogens or isolated from environmental sources, exhibited a limited degree of similarity, ranging between 15–51% at the nucleotide sequence level and having 21–51% of shared proteins (Figure 8). These include morphologically distinct phages (Figure 9) comprising of sub-clusters I1 (AbTJ and Ab105-1phi), L1 (RPH2R), L2 (TRS2), and the singleton TRS1.

Preliminary searches conducted with BLASTn, using the temperate *Acinetobacter* phage genomes, demonstrates the presence of similar prophage regions in a number of sequenced *A. baumannii* isolates, though at varying levels of coverage and sequence identity. Most isolates of *A. baumannii* appear to be poly-lysogens, with one study reporting a median of seven prophages across 177 analysed genomes [35]. In a larger study of 795 *A. baumannii* genomes, Costa et al. identified 19 clusters of prophages with a genome identity above 50% [36]. However, the impact of prophage carriage upon virulence, fitness, and metabolism in *Acinetobacter* lysogens has yet to be investigated in detail.

### 3.8. New Strains in the Genus Lokivirus

**Figure 10:**
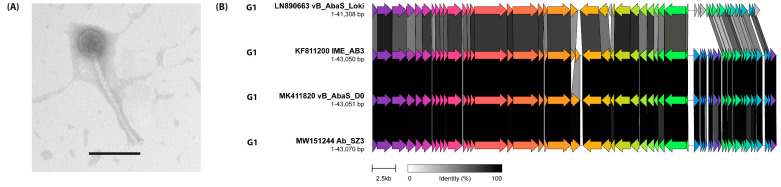
(**A**) Transmission electron micrograph of *Acinetobacter* phage Loki. The scale bar represents 100 nm. (**B**) Comparative genome alignment of phages comprising sub-cluster G1. Phage genomes are presented alongside their designated sub-cluster, accession number, name and genome length. Coding sequences are represented by arrows, coloured to reflect homologous groups identified by Clinker, and are linked by grey bars shaded to represent the percentage amino acid identity, as indicated in the legend.

The genus *Lokivirus* is expanded to include an additional two strains of IME_AB3 D0 and Ab_SZ3. Cluster G currently contains five siphoviruses that possess modular, syntenic genomes of between 41.3 and 43 kb and share 43 core protein groups, which represent the gene products involved in genome replication, transcriptional regulation, and virion structural proteins (Figure 10).

The two species are distinguished solely by differences in the structure of the endolysin and by the complement of genes in the predicted early expression module. Lipooligosaccharide has been implicated as the target cell surface receptor for the infection of *A. baumannii* ATCC 17978 by Loki as a stop mutation in *lpxA*, which is involved in the biosynthesis of lipid A, abolishes adsorption [37].

### 3.9. Phages DMU1 and SH-Ab15497 Comprise a New Genus of Siphoviruses

**Figure 11:**
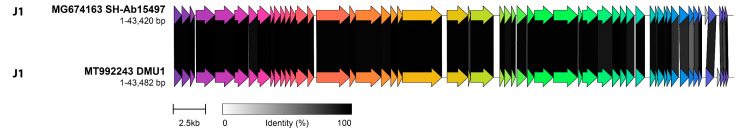
Comparative genome alignment of phages comprising sub-cluster J1. Phage genomes are presented alongside their designated sub-cluster, accession number, name and genome length. Coding sequences are represented by arrows, coloured to reflect homologous groups identified by Clinker, and are linked by grey bars shaded to represent the percentage amino acid identity, as indicated in the legend.

We propose the creation of a new cluster to group phages DMU1 and SH-Ab15479, which is supported by a nucleotide similarity of 92.6% and a total of 98.1% proteins shared across the two genomes. Both phages exhibit a Bradley B1 siphovirus morphology and a 43.4 kb genome with 53 gene products. Of the 53 core proteins, the majority (41 proteins) are only found within this sub-cluster. The 11 proteins shared with other *Acinetobacter* phages include seven virion structural proteins and four hypothetical proteins (Figure 11).

### 3.10. Singleton Phages

#### 3.10.1. Singletons Presley and XC38 Belong to the Family *Schitoviridae*

**Figure 12:**
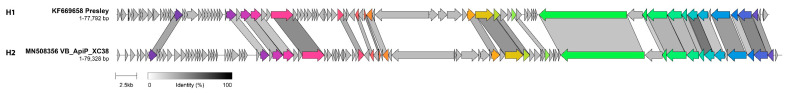
Comparative genome alignment of *Acinetobacter* phages classified within the family *Schitoviridae*. Phage genomes are presented alongside their designated sub-cluster, accession number, name and genome length. Coding sequences are represented by arrows, coloured to reflect homologous groups identified by Clinker, and are linked by grey bars shaded to represent the percentage amino acid identity, as indicated in the legend.

Presley and XC38 are the only two representatives of the family *Schitoviridae* isolated to date that infect the species *A. baumannii* and *A. pittii*. The family is characterised by a podovirus morphology with members encapsulating linear genomes of 59–90 kb flanked by direct terminal repeats. A total of 17 proteins are conserved across all members of the family, including the characteristic virion-associated and two-subunit RNA polymerases [38]. Based on their limited degree of nucleotide similarity (11%), Presley and XC38 are classified into two separate genera, *Presleyvirus* and *Xceevirus*. A total of 28 proteins (33.7%) are shared between these two species, and, with the isolation of additional representative species, may support the creation of a new subfamily within the *Schitoviridae* (Figure 12).

#### 3.10.2. Singletons BS46 and B9 Represent a New Subfamily of Myoviruses

**Figure 13:**
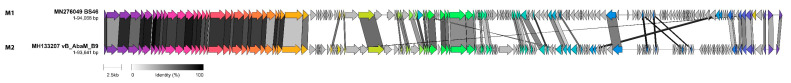
Comparative genome alignment of phages BS46 and B9. Phage genomes are presented alongside their designated sub-cluster, accession number, name and genome length. Coding sequences are represented by arrows, coloured to reflect homologous groups identified by Clinker, and are linked by grey bars shaded to represent the percentage amino acid identity, as indicated in the legend.

The myovirus BS46 was originally isolated in 1991 from sewage and used in an early assessment of the phage treatment of *A. baumannii* in a murine model [39]. BS46 was subsequently sequenced 25 years later, revealing a 94 kb linear genome with 176 predicted ORFs and three tRNAs [40]. B9 possesses a similarly sized genome of 93.6 kb and 168 predicted ORFs. Both B9 and BS46 form plaques surrounded by an opaque halo of reduced turbidity and encode structural proteins with capsule-specific depolymerase activity [40,41]. BS46 and B9 exhibited a 35% nucleotide sequence similarity and share 79 (51%) ORFs across the genome replication, virion structure, and assembly modules (Figure 13). Given the degree of similarity exhibited between these two phages, we propose that they are classified as separate genera within a new subfamily.

#### 3.10.3. Singleton ABPH49 Is a New Species of *Vequintavirinae*

ABPH49 is the only representative of the subfamily *Vequintavirinae* known to infect *A. baumannii* and shows little sequence similarity to the existing phages in the extant sequence database. The relationship of ABPH49 to the *Vequintavirinae* is revealed by TBLASTX, where the genome exhibits approximately 40% coverage and ~82% identity to members of the *Cetrevirus*. The 149.9 kb ABPH49 genome encodes 246 unique proteins and a further 32 that are shared with other *Acinetobacter* phages.

#### 3.10.4. Singleton SH-Ab 15599 Is a New Species of *Ackermannviridae*

The singleton phage SH-Ab 15599 has an A1 myovirus morphology and a genome of 143.2 kb [42], where 127 of the 174 predicted ORFs are unique among the *Acinetobacter* phages. The vContact2 network analysis places this phage within a cluster formed by the family *Ackermannviridae*. While SH-Ab15599 shows no discernible nucleotide sequence similarity to these phages, searches using TBLASTX reveals some similarity to members of the genus *Kuttervirus* (~30% coverage and 45% identity). Like other members of the *Ackermanniviridae*, SH-Ab 15599 exhibits the distinctive terminal tail morphology of branched tail fibers, in which each branch is formed by a single tailspike, suggesting that it may represent a new genus within this family.

#### 3.10.5. Arae Is a Genomic Singleton

**Figure 14:**
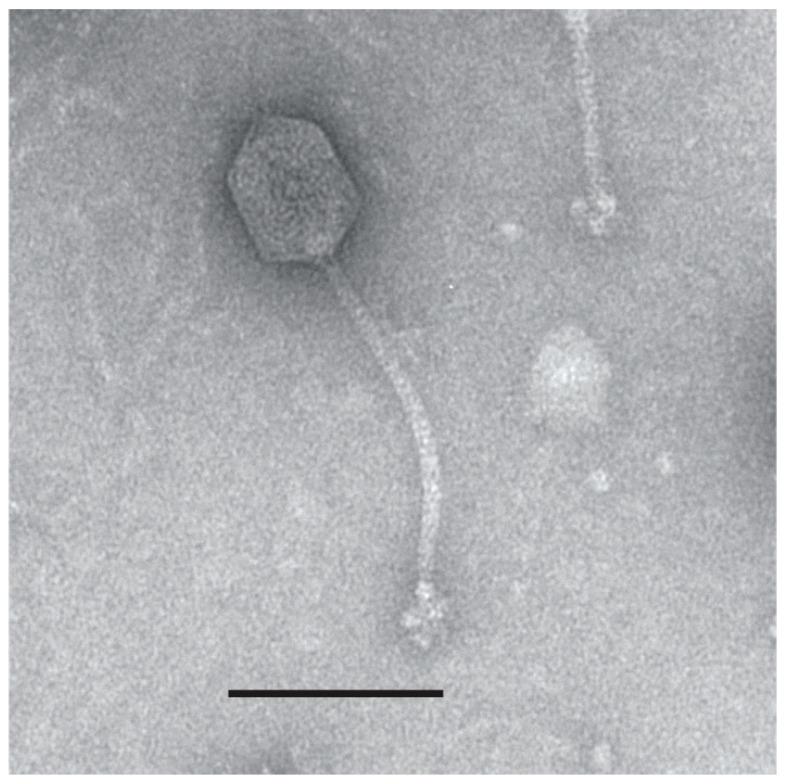
Transmission electron micrograph of phage Arae, negatively stained with 2% *w/v* uranyl acetate. The scale bar represents 100 nm.

Arae is a Bradley morphotype B2 siphovirus with a club-like distal tail structure that was isolated from raw sewage after enrichment with the *A. baumannii* clinical strain UKA9 (Figure 14). Arae encapsulates a 49.7 kb genome, which, at the time of writing, represents a genomic orphan with no phages showing similarity in the extant nucleotide sequence database. A total of 55 of the 66 predicted ORFs are unique and only 11 proteins are shared with other *Acinetobacter* phages.

#### 3.10.6. ME3, TRS5 and BFG Are Unique ‘Jumbo’ Singleton Phages of *Acinetobacter*

Recent metagenomics work has demonstrated that ‘jumbo’ phages, defined as those with genomes greater than 200 kb in size, are widely distributed in the environment [43]. To date, three jumbo phages have been isolated that infect *Acinetobacter* species, ME3, TRS5, and BFG.

**Figure 15:**
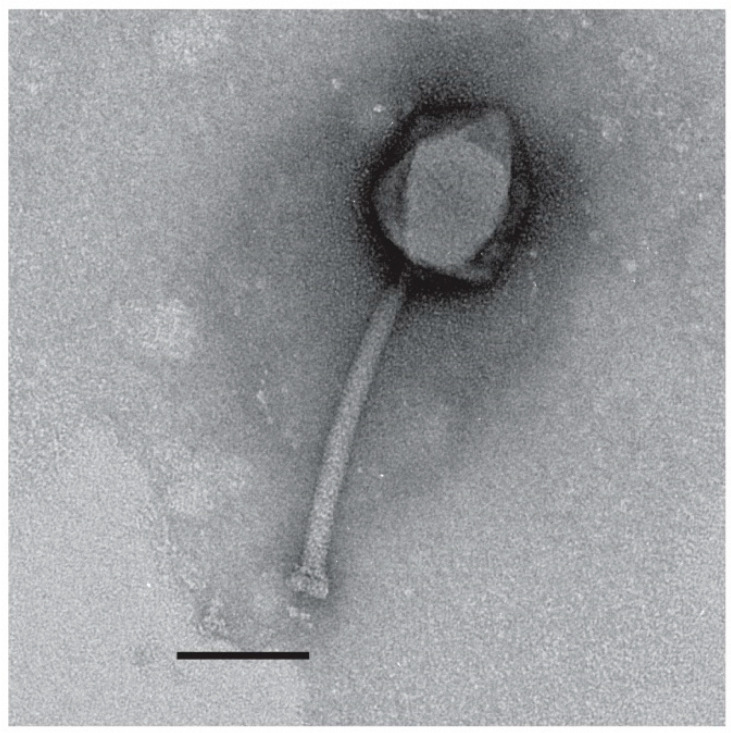
Transmission electron micrograph of the jumbo phage TRS5, stained with 2% *w/v* uranyl acetate. The scale bar represents 100 nm.

ME3 was originally isolated from wastewater treatment effluent and encapsulates a 234.9 kb genome with 326 ORFs and 4 tRNAs. ME3 remains a genomic orphan, exhibiting no significant sequence similarity to the phages in the extant sequence database and is classified as the only member of the genus *Metrivirus*. The original isolate of ME3 has since been lost (C. Buttimer, personal communication). TRS5 and BFG are morphologically identical myoviruses (Figure 15) that were isolated from activated sludge and raw sewage from the same water treatment plant and propagate on *A. baylyi* ADP1 and *A. baumannii* UKA17, respectively. Despite the apparent morphological similarity, the genomes exhibit only a 17% nucleotide similarity. TRS5 and BFG encapsulate genomes of 371.5 and 378.1 kb with 661 and 666 predicted ORFs and ≥20 tRNAs, respectively. These phages do not exhibit the modular organisation characteristic of smaller phage genomes. Instead, for TRS5 and BFG, ORFs appear to be organised into operons, which are flanked by promoters and rho-independent terminators. Despite the low level of sequence similarity, these two phages share a total of 214 proteins. A significant proportion (>60%) of the genes encoded by these three phages are classified as unique, and the majority cannot be assigned predicted functions using current bioinformatics tools, suggesting that these phages represent distinct evolutionary lineages.

### 3.11. Select Features of Acinetobacter Phage Proteins

The selection and development of phages for the knowledge-based application of phage therapy relies upon phenotypic as well as genomic data. Aside from the well-established criteria of avoiding phages that are temperate (unless genetically modified to prevent integration) and those that carry putative virulence factors or antibiotic resistance genes, the prediction of phenotype from genomic data—such as host specificity, resistance to host defense systems, and lysis mechanisms—may help to inform the selection of appropriate phages for clinical use or formulation within phage cocktails. To achieve this, it is vital to assess the biological role of *Acinetobacter* phage-derived proteins. Using the annotated pan-genome analysis, gene products involved in nucleotide modification, lysis, and virion structural depolymerases are discussed.

#### 3.11.1. Nucleotide Modification Systems

Our analysis indicates that 39 (29%) of the *Acinetobacter* phages encode methyltransferases. There are 13 phages that possess adenine-specific methyltransferases, while cytosine-specific methytransferases appear to be more widespread and are present in 28 genomes. While most encode a single methyltransferase, some phages possess two or more. For example, the phages BFG, TRS5, and 5W encode both adenine- and cytosine-specific methyltransferases. BS46 and BFG are each predicted to code for two distinct adenine-specific methyltransferases that exhibit little sequence similarity.

The InterProScan and HH-suite results provided evidence for the presence of further gene products potentially involved in nucleotide modifications, including a 7-deazaguanine modification pathway in the jumbo phage TRS5. Recent work has shown the presence of a number of different 7-deazaguanine derivatives in phage genomes, including 2′-deoxy-7-cyano-7-deazaguanosine (dPreQ_0_), 2′-deoxy-7-amido-7-deazaguanosine (dADG), 2′-deoxy-7-aminomethyl-7-deazaguanosine (dPreQ1), and 2′-deoxyarchaeaosine (dG^+^) [44]. TRS5 encodes homologues to *queC, queD, queE*, *queF,* and *dpdA* in a localised cluster of genes. A distally located *folE*-like GTP cyclohydrolase is also present in the genome.

SH-Ab15599 codes for two proteins containing an Alpha-glutamyl/putrescinyl thymine pyrophosphorylase clade 3 domain (InterPro: IPR041271) that is predicted to be involved in the catalysis of the hyper-modified bases alpha-glutamylthymine, putrescinylthymine, 5-(2-aminoethoxy)methyluridine, or 5-(2-aminoethyl)uridine. These types of modified pyrimidines have been shown to occur in several phage genomes, including ΦW-14 [45], SP10 [46], ViI [47], and M6 [47].

The sub-cluster K1 phages SH-Ab 15497 and DMU1 each encode a gene product with a predicted adenylosuccinate synthetase activity (IPR042109; group 911) that may be involved in purine biosynthesis. However, whether these gene products are involved in the synthesis of modified bases, as has been observed for the Cyanophage S-2L that incorporates 2,6-aminoadenine in place of adenine [48], remains to be determined.

In phages T2, T4, and T6, the genomic DNA contains 5-hydroxymethylcytosine (^hm5^C), which is further modified by glycosylation to yield glucosyl-5-methylcytosine. This process is catalysed by dCMP hydroxylmethytransferase (gene 42 in T4), yielding a hydroxymethylated cytosine monophosphate, which is then converted by T4 dNMP kinase to the diphosphate form. Following replication, ^hm5^C is glucosylated by one of two DNA glucosyltransferases that produces an alpha or beta linkage between glucose and ^hm5^C [48]. While homologues to a dCMP hydroxymethylase and T4 dNMP kinase were identified within the *Twarogvirinae* (Cluster A), we were unable to predict the presence of either alpha- or beta glucosyltransferases using bioinformatics approaches, so whether these phages possess glucosylated DNA remains unknown.

#### 3.11.2. Lysis Cassettes

The *Acinetobacter* phages have highly conserved endolysin and holin genes, with the most conserved present in 64 and 50 phages, respectively. About 81% are predicted to encode canonical holin-globular endolysins, 17% holin-modular endolysins, and 2% pinholin-SAR endolysins. The rare modular endolysin architecture is present only in the members of clusters B, I, F, and G, as well as in the singletons (e.g., 5W). In all cases, these proteins display an N-terminal Glyco_hydro_108 (PF05838.14) catalytic and a C-terminal PG_binding_3 (PF09374.12) domain. The Signal-arrest-release (SAR) endolysins are exclusively present in cluster K, having an N-terminal transmembrane domain and a lysozyme catalytic domain (PF00959). Surprisingly, we did not identify proteins with spanin functions needed to disrupt the host outer membranes, suggesting that lysis cassettes lacking spanins may be a feature of *Acinetobacter* phages, which may have adopted an alternative mechanism to disrupt the outer membranes.

#### 3.11.3. Virion Structural Polysaccharide Depolymerases

Virion structural depolymerases appear to be highly prevalent among the *Acinetobacter* viruses. From the 134 *Acinetobacter* phage genomes analysed, depolymerases were identified in 83 phages (62%), indicating that the remaining phages (38%) recognize hosts without enzymatic activity, either via LPS or outer membrane proteins. The most conserved depolymerase gene is shared by only 18 phages, demonstrating the vast diversity of these proteins. Although prevalent, depolymerase genes are exclusively located in clusters containing viruses with genomes of less than 90 kb (B, D, E, I, J, K, L, and M), and three singletons (SH-Ab15599, TRS1, and 5W). All members of these clusters encode depolymerases with no exceptions. For instance, while *Autographiviridae* viruses (cluster E) have genomes around 40 kb, clusters B, D, I, J, K, L, and M contain viruses with myovirus and siphovirus morphotypes that also have genome sizes ranging between 40 to 90 kb. By homology, 73 phages (54.5%) contain capsular depolymerases, with the remaining 10 (7.5%) encoding esterases—a class recently characterized in the *Acinetobacter* phage Aristophanes [49]. With the exception of the singleton SH-AB_15599, which codes for two adjacent capsular depolymerases, all viruses analysed here are predicted to code for a single depolymerase.

## 4. Discussion

As for many other host bacterial genera and species, there is significant diversity in the phages infecting *Acinetobacter* species. Since 2018, the number of sequenced *Acinetobacter* phage genomes available in the INSDC has expanded from 37 to 139. This number is expected to continue to increase given the importance of *A. baumannii* as a priority critical ESKAPE pathogen and the expanding interest in phage therapy. Our results demonstrate that these phages can be classified using nucleotide sequence identity, shared orthologous genes, and genomic organisation. The *Acinetobacter* phages are grouped into eight clusters (subfamilies), 38 subclusters (genera), and eight singleton genomes (single species genera). While the majority of core protein groups identified for each cluster represent genes that are coding for key products in genome replication, lysis, and virion structure and assembly, some core genes and numerous accessory protein groups of unknown function are located within the predicted early genome modules.

While the number of sequenced genomes of phages infecting *Acinetobacter* species has increased, little is still known about the underlying biology governing the subversion of host transcription, translation, and metabolism that facilitates a productive infection. Genes expressed early in phage infection have been associated with host takeover [50]. These early gene products have received increased attention for the identification and exploitation of novel antibacterial proteins and mechanisms of action [51]. Advances in RNA sequencing have allowed the temporal expression of phage genes across different timepoints in the infection cycle for a number of different phage–host combinations, including *Yersinia* [52], *Vibrio* [53], and *Pseudomonas* [54,55]. However, few early genes have been identified in *Acinetobacter* phage genomes. Instead, this status is often ascribed due to the position of genes within a genomic module. To date, only a single study has reported transcriptome data for infection in the *A. baumannii* strain AB1 with phages Abp1 (Cluster E) and AB1 (Cluster B) [56]. Given the diversity observed in the protein-coding genes of the *Acinetobacter* phages, it is likely that a variety of distinct mechanisms play a role in the early infection of host cells, some of which may be dependent upon the physiological status of the host cell (e.g., under conditions of nutrient limitation). A key challenge to furthering our understanding of productive infection in the *Acinetobacter* phages is to determine the functions of such early proteins, and to elucidate their roles and effects in the context of gene regulation, transcription, protein-protein interactions, and host cell metabolism. In addition to providing potential avenues for the identification of novel antibacterial mechanisms, such work might accommodate the need for tools in the genetic analysis and manipulation of this important pathogen.

Infections caused by carbapenem-resistant *A. baumannii* will continue to be of significant concern in the coming years. The WHO has highlighted that the current clinical pipeline still lacks novel antibiotics targeting this priority pathogen, and that those in development may be affected by cross-resistance given that most are derivatives of existing classes of antibiotics [57,58]. Given the increased interest in the application of phage therapy and use of phage-derived proteins, such as depolymerases and endolysins, it will be necessary to develop a platform that captures the important phenotypic features of individual phages, including host range and adsorption specificity, one step growth characteristics, and stability data, to supplement genomic information.

Many phages have adapted to encode tail fiber or tail spike proteins with enzymatic (depolymerase) activity to recognize and degrade the polysaccharides that decorate the bacterial cell surface [59,60]. The diversity of the capsular polysaccharides incurs a constant selective pressure on phages to evolve depolymerases with different specificities [61]. Current knowledge has shown that phage-derived depolymerases may degrade lipopolysaccharide (LPS) [62], capsule polymers [63], or modify surface polysaccharides through deacetylation [64]. However, only a small fraction of *Acinetobacter* phages have been reported to show depolymerase activity, and, to date, act predominantly upon capsular polysaccharides [41,63,65,66].

Capsular depolymerases have been applied as a method to reduce *A. baumannii* virulence [65,67]. By stripping the cell surface capsular coats, depolymerases have been shown to render *A. baumannii* cells less virulent and more susceptible to the host immune system. As such, there is particular interest in understanding phage specificity for *Acinetobacter* capsular polysaccharides. To date, phages that are specific to 16 different capsular types of *A. baumannii* (K1–K2, K9, K19, K27, K32, K37–K38, K44–K45, K48, K87, K89, K91, K93, and K116) have been described [63,65,66,68,69,70]. Here, we have shown that there is a large untapped source of depolymerases for *Acinetobacter* capsule types, which could be used as anti-virulence or anti-biofilm agents against *A. baumannii* infections. Elucidating the tertiary structures of the additional depolymerases could provide a structural framework by which to produce enzymes with broader specificity, or allow the engineering of phages with altered host specificity, as peformed in *Klebsiella* phages [71].

Phages of the class *Caudoviricetes* encode lysis cassettes to burst the host cell wall at the end of their lytic cycle in order to release progeny virions. Phages infecting Gram-negative bacteria are generally described with a holin–endolysin–spanin system. Holins act first to permeabilise the cytoplasmatic membrane to endolysins that then degrade the rigid peptidoglycan layer, resulting in osmotic lysis and cell death. Spanins are also used by phages, either as two-component or unimolecular proteins, to disrupt the outer membranes in this final step [72]. It was surprising to observe a lack of predicted spanins in the *Acinetobacter* phages. It is possible that these phages are using endolysins to interact with the outer membrane in the final step of the lytic cycle. A growing body of evidence has shown the ability of recombinant *Acinetobacter* phage endolysins to naturally degrade cells after exogenous application due to the presence of highly positively charged C-terminal domains with predicted amphipathic helical structures [73,74]. Another possibility might be as reported in the coliphage φKT phage, which encodes an independent protein-encoding gene that disrupts the membrane using a mechanism similar to antimicrobial peptides [75].

Restriction–modification (R–M) systems are perhaps the most well characterized resistance mechanisms against foreign DNA. These systems are widespread among prokaryotes and are present in 90% of bacterial genomes [76]. R–M systems are categorized into four main types: type I, II, III, and IV. Type II systems, consisting of a restriction endonuclease and cognate DNA adenine or cytosine methyltransferase, are the most abundant and the best studied so far [77]. In type II systems, the restriction endonuclease and methyltransferase both recognize the same specific target sequence. In the absence of methylation at the recognition site, the restriction enzyme cleaves the DNA. This acts as a defense mechanism against foreign DNA, such as phages. To counteract the bacterial R–M defense systems, phages have evolved several different strategies that include the synthesis of methyltransferases, production of anti-restriction proteins, and the use of a variety of modified bases [78]. Nucleotide modification appears to be relatively widespread among the *Acinetobacter* phages. A total of 39 phages were predicted to encode adenine or cytosine specific methyltransferases and three phages had gene products that may point to the presence of other types of modification, which includes a 7-deazaguanine derivative. Considering that methylation has been shown to have additional functions in bacteria, including roles in DNA replication, DNA repair, and regulation of transcription [79], it is possible that the presence of modified nucleosides in phage genomes may play a wider role in the infection cycle than just defence against restriction–modification systems. To date, no studies have specifically addressed the presence of modified bases in *Acinetobacter* phage genomes.

While the numbers and diversity of *Acinetobacter* phage genomes in the INSDC has increased, we still know little about the underlying biology around both the mechanisms of productive infection by lytic phages and the effects of prophage carriage upon the lysogen in this host genus. While this work provides an updated framework to aid in the annotation and classification of newly isolated *Acinetobacter* phages, it is clear that more applied research is required to supplement the information derived from the in silico analysis presented here.

## Figures and Tables

**Table 1:** Diversity of *Acinetobacter* phage genomes, composed of 8 clusters, 38 sub-clusters, and 8 singletons. Core, accessory, and unique protein groups identified within each cluster and sub-cluster are presented. Clusters corresponding to subfamilies are presented in bold type. Numbers in parentheses refer to protein groups found exclusively within the designated cluster. Ratified ICTV taxa are presented in italics, while proposed subfamily and genus names are presented as roman text within quotations.

Cluster	Taxonomy	Representative Phage	No. of Genomes	No. of Core Protein Groups	No. of Accessory Protein Groups	No. of Unique Proteins
**A**	***Twarogvirinae***	**-**	**25**	**107 (81)**	**255 (225)**	**252**
A1	*Zedzedvirus*	ZZ1	2	241 (52)	N/A	4
A2	*Lasarusvirus*	Lazarus	12	218 (11)	32 (22)	2
A3	*Hassadvirus*	PhT2	7	227 (13)	26 (17)	3
A4	*Lasallevirus*	Acj61	1	N/A	N/A	46
A5	*Acajnonavirus*	Acj9	1	N/A	N/A	46
A6	“Freretvirus”	Ac42	1	N/A	N/A	74
A7	“Audubonvirus”	133	1	N/A	N/A	77
**B**	**“Zhukovskyvirinae”**	**-**	**17**	**23 (22)**	**117 (100)**	**63**
B1	“Solivirus”	phiAC-1	1	N/A	N/A	33
B2	“Fengtaivirus”	IME-AB2	8	56 (4)	36 (4)	6
B3	“Shapingbavirus”	AbP2	5	58 (1)	42 (33)	5
B4	*Obolenskvirus*	AP22	1	N/A	N/A	3
B5	“Wenzhouvirus”	AB1	1	N/A	N/A	8
B6	“Dongdavirus”	IME284	1	N/A	N/A	9
**C**	**“Astridvirinae”**	**-**	**8**	**69 (42)**	**117 (106)**	**88**
C1	“Acibelquatrovirus”	Acibel004	1	N/A	N/A	58
C2	“Powislevirus”	phiAbaA1	1	N/A	N/A	15
C3	*Saclayvirus*	TAC1	6	129 (97)	32 (28)	15
D1	“Caradocvirus”	AM24	2	115 (87)	N/A	45
E *	***Beijernickvirinae***	**-**	**49**	**21 (20)**	**57 (45)**	**69**
E1	“Aristophanvirus”	Aristophanes	1	N/A	N/A	15
E2 *	*Friunavirus*	Fri1	45	27 (0)	70 (61)	15
E3	*Pettyvirus*	Petty	1	N/A	N/A	12
E4	*Daemvirus*	Acibel007	1	N/A	N/A	13
E5	“Lisbonvirus”	F1245-05	1	N/A	N/A	14
**F**	**“Junivirinae”**	**-**	**7**	**11 (2)**	**87**	**96**
F1	“Shemyakinvirus”	AM106	1	N/A	N/A	16
F2	“Bogotavirus”	Ab11510-phi	1	N/A	N/A	8
F3	*Vieuvirus*	Bphi-B1251	2	44 (12)	N/A	17
F4	“Breacavirus”	RPH5R	1	N/A	N/A	19
F5	“Geihvirus”	Ab105-2phi	1	N/A	N/A	18
F6	“Reipivirus”	Ab105-3phi	1	N/A	N/A	18
G1	*Lokivirus*	Loki	4	43 (38)	10 (9)	5
**H**	***Schitoviridae***	**-**	**2**	**28 (28)**	**N/A**	**104**
H1	*Presleyvirus*	Presley	1	N/A	N/A	54
H2	*Xceevirus*	XC38	1	N/A	N/A	50
I1	“Xubiasvirus”	Ab105-1phi	2	58 (33)	N/A	4
J1	“Stillvirus”	DMU1	2	53 (41)	N/A	2
K1	“Haartmanvirus”	fEg-Aba01	3	47 (32)	2 (1)	0
**L**	**“Grainevirinae”**	**-**	**2**	**21 (18)**	**N/A**	**26**
L1	“Corvusvirus”	RPH2R	1	N/A	N/A	14
L2	“Boudicavirus”	TRS2	1	N/A	N/A	14
**M**	**“Soothillvirinae”**	**-**	**2**	**79 (53)**	**N/A**	**129**
M1	“Bragavirus”	B9	1	N/A	N/A	56
M2	“Bathrowvirus”	BS46	1	N/A	N/A	73
Singleton	*Metrivirus*	ME3	1	N/A	N/A	261
Singleton	“Gogmagogvirus”	TRS5	1	N/A	N/A	405
Singleton	“Comoranvirus”	BFG	1	N/A	N/A	422
Singleton	“Lianhecunvirus”	5W	1	N/A	N/A	22
Singleton	“Fengtaivirus”	ABPH49	1	N/A	N/A	246
Singleton	“Camillusvirus”	TRS1	1	N/A	N/A	23
Singleton	“Jiatongvirus”	SH-Ab 15599	1	N/A	N/A	127
Singleton	“Lucanusvirus”	Arae	1	N/A	N/A	55

* Number of coding sequences calculated after the exclusion of the partial genome of phage AB3.

## Data Availability

Not applicable.

## Supplementary Materials

The following are available online at https://www.mdpi.com/article/10.3390/v14020181/s1, Figure S1: VIRIDC analysis of 139 *Acinetobacter* phages, Table S1: *Acinetobacter* bacteriophage genomes deposited in GenBank on the 21 January 2021, File S1: Pan-genome analysis and annotation of proteins encoded by 139 *Acinetobacter* phages.
