# Supplementary material for: Genomic Diversity of Bacteriophages Infecting the Genus Acinetobacter
Source: Viruses. 2022 Jan 19;14(2):181. doi: 10.3390/v14020181 (PMC8878043; doi:10.3390/v14020181)
Supplement: Supplementary file 1 [file viruses-14-00181-s001.zip › Table_S1.pdf]

Supplementary Table S1. *Acinetobacter* bacteriophage genomes deposited in GenBank on the 21st of January 2021. * Partial genome; #Number of coding sequences in the annotated sequence record; $Genomes submitted without annotation.

Cluster	Sub-cluster	Accession	Phage Name	Propagating Host	Country of Isolation	Genome size (bp)	%G+C	Number of CDS#	Reference
A	A1	HQ698922	ZZ1	*A. baumannii* AB09V	China	166,682	34.41	256	[1,2]
	A1	MK240351	Henu6	*A. baumannii* ATCC19606	China	166,890	34.33	234	-
	A2	MH165274	AM101	*A. baumannii* LUH 3712	Russia	166,487	36.71	250	-
	A2	MH460829	vB_ApiM_fHyAci03	*A. pittii*	Finland	165,975	36.76	247	[3]
	A2	MH713599	KARL-1	*A. baumannii*	Germany	166,560	36.79	253	[4]
	A2	MN648195	Konradin	*A. baumannii* MAR-610	Russia	166,210	36.75	249	-
	A2	MN662249	Stupor	*A. baumannii* RES-2281	Russia	164309	36.83	245	-
	A2	MN709128	Berthold	*A. baumannii* LUH3713	Russia	165,927	36.71	250	-
	A2	MN723850	Apostate	*A. baumannii* KZ1100	Russia	164,746	36.75	244	-
	A2	MN732883	Kimel	*A. baumannii* RES-3023	Russia	167,922	36.73	252	-
	A2	MN782535	Lazarus	*A. baumannii* MAR15-4162	Russia	165,151	36.76	247	-
	A2	MT385367	Abraxis	*A. baumannii* MAR16-1722	Russia	166559	36.78	249	-
	A2	MT409116	Octan	*A. baumannii* RES-241	Russia	166760	36.72	247	-
	A2	MT741943	Meroveus	*A. baumannii* RES-241	Russia	167,683	36.72	248	-
	A3	MK278860	AbTZA1	*A. baumannii*	Israel	168,223	36.35	253	-
	A3	MN864865	vB_AbaM_PhT2	*A. baumannii*	Thailand	166,330	36.37	255	-
	A3	MT949699	Maestro	*A. baumannii* TP1	USA	169,176	36.33	264	-
	A3	MW388001	Minot	*A. baumannii*	USA	167,029	36.33	248	-
	A3	MW388002	Mokit	*A. baumannii*	USA	167,638	36.32	250	-
	A3	MW388003	Melin	*A. baumannii*	USA	167,969	36.33	252	-
	A3	MW388004	Morttis	*A. baumannii*	USA	169,150	36.33	259	-
	A4	GU911519	Acj61	*A. johnsonii*	USA	164,093	39.01	241	[5]
	A5	HM004124	Acj9	*A. johnsonii*	USA	169,947	40.03	253	[5]
	A6	HM032710	Ac42	*Acinetobacter* sp. (HER 1422)	Canada	167,716	36.37	255	[5]
	A7	HM114315	133	*A. johnsonii* (HER 1423)	Canada	159,801	39.67	257	[6,7]

B	B1	JX560521	phiAC-1	*A. soli* KZ-1	South Korea	43,216	38.48	82	[8]
	B2	JX976549	IME-AB2	*A. baumannii* MDR-AB2	China	43,665	37.5	82	[9,10]
	B2	KJ817802	YMC-13-01-C62 (Bφ-C62)	*A. baumannii* YMC/13/01/C62	South Korea	44,844	37.6	84	[11]
	B2	KP861229	YMC11/12/R2315 (Bφ-R2315)	*Acinetobacter* sp.	South Korea	44,846	37.59	85	[12]
	B2	KP861231	YMC11/12/R1215 (Bφ-R1215)	*Acinetobacter* sp.	South Korea	44,866	37.59	85	[12]
	B2	KU510289	LZ35	*A. baumannii*	China	44,885	38.36	83	[13]
	B2	MH853786	vB_AbaM_IME285	-	China	45,063	37.87	85	[14]
	B2	MN516421	Bphi-R2919	*A. baumannii* YMC18/02/R2919	Korea	44,227	37.77	80	-
	B2	MN516422	Bphi-R1888	*A. baumannii* YMC17/03/R1888	Korea	44,590	37.87	78	-
	B3	KY670595	WCHABP12	*A. baumannii*	China	45,415	37.58	88	[15]
	B3	KY829116	WCHABP1	*A. baumannii*	China	45,888	37.63	89	[15]
	B3	MF346584	AbP2	*A. baumannii* AB2	China	45,373	37.84	88	-
	B3	MH853788	vB_AbaM_IME512	-	China	46,499	37.56	91	-
	B3	MN166083	Abp9	*A. baumannii*	China	44,820	37.69	81	[16,17]
	B4	HE806280	AP22	*A. baumannii* 1053	Russia	46,387	37.74	89	[18-20]
	B5	HM368260	AB1	*A. baumannii* KD311	China	45,159	37.69	88	[21,22]
	B6	MH853787	vB_AbaM_IME284	-	China	43,557	38.3	84	-
C	C1	KJ473422	Acibel004	*A. baumannii* 070517/0072	Belgium	99,730	37.27	156	[23,24]
	C2	KJ628499	vB_AbaM_phiAbaA1	*A. baumannii* Acb8/09	Poland	104,906	37.77	165	-
	C3	MH746814	vB_AbaM_B09_Aci05	*A. baumannii* Paris B09	Cote d'Ivoire	102,789	37.21	160	[25]
	C3	MH800198	vB_AbaM_B09_Aci01-1	*A. baumannii*	Cote d'Ivoire	103,628	37.19	163	[25]
	C3	MH800199	vB_AbaM_B09_Aci02-2	*A. baumannii*	Cote d'Ivoire	104,354	37.18	171	[25]
	C3	MK170160	TAC1	*A. baumannii*	Pakistan	101,770	37.49	163	[26]
	C3	MT623546	Ab_121	*A. baumannii*	China	102,499	37.2	159	[27]
	C3	MT188223	vB_AbaM_D22	*Acinetobacter sp.*	China	103,539	37.5	165	-
D	D1	KM672662	YMC13/03/R2096	*A. baumannii* YMC13/03/R2096	South Korea	98,170	37.04	162	[28]
	D1	KY000079	AM24	*A. baumannii*	Russia	97,139	37.25	146	[29,30]
E	E1	MT783706	Aristophanes	*A. baumannii* KZ-1098	Russia	43,505	42.53	46	[31]
	E2	HQ186308	phiAB1	*A. baumannii* M68316	Taiwan	41,526	39.09	46	[32]

	E2	JX658790	Abp1	A. baumanni AB1	China	42,185	39.15	57	[33-35]
	E2	KC311669	AB3*	*A. baumannii*	China	31,185	39.18	27	[36,37]
	E2	KR149290	Fri1	*A. baumannii* 28	Switzerland	41,805	39.29	54	[38]
	E2	KT339321	phiAB6	*A. baumannii* 54149	Taiwan	40,570	39.47	45	[39-41]
	E2	KT388102	vB_AbaP_PD-6A3	*A. baumannii*	China	41,563	39.92	48	[42]
	E2	KT388103	vB_AbaP_PD-AB9	*A. baumannii*	China	40,938	39.34	48	-
	E2	KT804908	IME200	*A. baumannii*	China	41,243	39.73	52	[43]
	E2	KY082667	SH-Ab15519	*A. baumannii*	China	40,493	39.46	51	[44]
	E2	KY268295	vB_AbaP_AS12	*A. baumannii* 1432	Russia	41,402	39.31	49	[38,45]
	E2	KY268296	vB_AbaP_AS11	*A. baumannii* 28	Russia	41,642	39.29	51	[45]
	E2	KY888680	WCHABP5	*A. baumannii* WCHAB1334	China	40,409	39.38	47	-
	E2	MF033347	vB_AbaP_B1	*A. baumannii*	Portugal	40,879	39.14	51	[46]
	E2	MF033348	vB_AbaP_B3	*A. baumannii*	Portugal	40,598	39.25	49	[46]
	E2	MF033349	vB_AbaP_B5	*A. baumannii*	Portugal	41,608	39.31	53	[46]
	E2	MF033350	vB_ApiP_P1	*A. pittii* NIPH 76	Portugal	41,208	39.2	49	[46]
	E2	MF033351	vB_ApiP_P2	*A. pittii* NIPH 76	Portugal	41,514	39.33	54	[46]
	E2	MG459218	SWH-Ab-1	*A. baumannii*	China	41,567	39.42	50	
	E2	MH042230	vB_AbaP_D2	*A. baumannii*	China	39,964	39.23	47	[47,48]
	E2	MH763831	vB_AbaM_B09_Aci08	*A. baumannii* Paris B09	Cote d'Ivoire	42,067	39.28	53	[25]
	E2	MH800200	vB_AbaP_46-62_Aci07	*A. baumannii*	Cote d'Ivoire	42,330	39.14	52	[25]
	E2	MK089780	vB_AbaP_APK14	*A. baumannii* AB5256	Russia	41,767	39.44	55	-
	E2	MK257719	vB_AbaP_APK2	*A. baumannii* ACICU	Russia	41,476	39.24	50	[49]
	E2	MK257720	vB_AbaP_APK2-2	*A. baumannii* ACICU	Russia	41,426	39.38	50	-
	E2	MK257721	vB_AbaP_APK93	*A. baumannii* B11911	Russia	41,668	39.38	50	[49]
	E2	MK257722	vB_AbaP_APK32	*A. baumannii* LUH5549	Russia	41,142	39.31	52	[49]
	E2	MK257723	vB_AbaP_APK37	*A. baumannii* NIPH146	Russia	41,981	39.16	51	[49]
	E2	MK278859	AbKT21phiIII	*A. baumannii*	Israel	40,898	39.36	43	-
	E2	MN061582	vB_AbaP_IME546	*A. baumannii*	China	40,770	39.14	51	-
	E2	MN212906	vB_AbaP_D2M	*A. baumannii*	China	39,963	39.24	43	-

	E2	MN433707	vB_AbaP_PMK34	*A. baumannii* MK34	Egypt	41847	39.34	50	[50]
	E2	MN604238	vB_AbaP_APK44	*A. baumannii* NIPH70	Russia	41461	39.07	50	[49]
	E2	MN604239	vB_AbaP_APK87	*A. baumannii* LUH5547	Russia	42402	39.05	54	[49]
	E2	MN614471	vB_AbaP_APK48-3	*A. baumannii* APEX-294	Russia	46,499	39.23	54	-
	E2	MN651570	vB_AbaP_APK89	*A. baumannii* LUH5552	Russia	41198	39.4	52	[49]
	E2	MN807295	vB_AbaP_APK116	*A. baumannii* MAR303	Russia	41765	39.08	49	[49]
	E2	MT263719	vB_AbaP_AGC01	*A. baumannii*	Poland	41231	39.48	54	[51]
	E2	MT633129	Ab124	*A. baumannii* b1928040	China	40,471	39.5	47	-
	E2	MT741944	vB_AbaP_APK81	*A. baumannii* 36-1512	Russia	42,233	39.32	55	-
	E2	MW056501	vB_AbaA_fBenAci001	*A. baumannii*	Benin	40,535	39.24	50	-
	E2	MW056502	vB_AbaA_fBenAci002	*A. baumannii*	Benin	41,953	39.22	53	-
	E2	MW056503	vB_AbaA_fBenAci003	*A. baumannii*	Benin	40,556	39.23	47	-
	E2	MW345241	vB_AbaP_APK26	*A. baumannii* KZ-1098	Russia	41,097	39.26	54	-
	E2	MN294712	vB_AbaP_APK48	*A. baumannii* NIPH615	Russia	41,105	39.26	49	[49]
	E2	MN395291	vB_AbaP_IME546	*A. baumannii*	China	40,540	39.12	50	-
	E2	MW366783	Pipo	*K. pneumoniae*	USA	41,956	39.16	58	-
	E2	MW366784	Paty	*K. pneumoniae*	USA	41264	39.42	55	-
	E3	KF669656	Petty	*A. baumannii* AU0783	USA	40,739	42.19	45	[52,53]
	E4	KJ473423	Acibel007	*A. baumannii* 070517/0072	Belgium	42,654	41.7	53	[23,24]
	E5	HH777814	F1245/05	*Acinetobacter sp.*	Portugal	43,016	40.46	[$]	[54]
F	F1	MH115576	AM106	*A. baumannii* NIPH 329	Russia	52031	40.17	70	-
	F2	MT361972	Ab11510-phi	*A. baumannii* Ab11510	Mexico	50,916	38.87	76	[55]
	F3	JX403940	Bphi-B1251	*A. baumannii* YMC/09/02/B1251	South Korea	45,364	39.05	62	[56]
	F3	KP861230	YMC11/11/R3177	*Acinetobacter sp.*	South Korea	47,575	39.83	80	[57]
	F4	Unreleased	RPH5R	*A. baumannii* AYE	UK	52,112	38.37	71	-
	F5	KT588073	Ab105-3phi	*A. baumannii* Ab105	Spain	63,785	39.42	77	-
	F6	KT588075	Ab105-2phi	*A. baumannii* Ab105	Spain	52,456	39.43	93	[58]
G	G1	KF811200	IME-AB3	*A. baumannii*	China	43,050	45.48	57	-
	G1	LN890663	vB_AbaS_Loki	*A. baumannii* ATCC 17978	UK	41,308	44.35	51	[59]

	G1	MK411820	vB_AbaS_D0	*Acinetobacter sp.*	China	43,051	45.48	55	[47]
	G1	MW151244	Ab_SZ3	*A. baumannii*	China	43,070	45.62	56	-
H	H1	KF669658	Presley	*A. baumannii* M2	USA	77,792	37.77	95	[60]
	H2	MN508356	vB_ApiP_XC38	*A. pittii* ABC38	China	79,328	39.58	97	[61]
I	I1	KT588074	Ab105-1phi	*A. baumannii* Ab105	Spain	41,496	39.49	63	-
	I1	MK340941	AbTJ	*A. baumannii* MDR-TJ	China	42,670	39.32	62	[62]
J	J1	MG674163	SH-Ab 15497	*A. baumannii*	China	43,420	47.87	58	[63]
	J1	MT992243	DMU1	*A. baumannii* 19606	USA	43,482	47.83	59	[64]
K	K1	MT344103	fEg-Aba01	*A. baumannii* DSM 106838	Finland	33779	40.1	52	[65]
	K1	MT344104	fLi-Aba02	*A. baumannii* DSM 106838	Finland	35093	40.16	54	[65]
	K1	MT344105	fLi-Aba03	*A. baumannii* DSM 106838	Finland	34931	40.09	51	[65]
L	L1	Unreleased	RPH2R	*A. baumannii* AYE	UK	35,420	40.65	49	-
	L2	Unreleased	TRS2	*A. baumannii* UKA9	UK	35,157	40.81	52	-
M	M1	MH133207	vB_AbaM_B9	*A. baumannii*	Portugal	93,641	32.57	167	[66]
	M2	MN276049	BS46	*A. baumannii* AC54	UK	94,068	33.45	176	[38,67-69]
Singleton	-	KU935715	vB_AbaM_ME3	*A. baumannii* DSM 30007	Ireland	234,900	30.76	326	[70]
Singleton	-	Unreleased	BFG	*A. baumannii* UKA17	UK	378,110	32	675	-
Singleton	-	Unreleased	TRS5	*A. baylyi* ADP1	UK	371,547	29.66	673	-
Singleton	-	MT349887	5W	*A. baumannii*	China	43032	39.84	61	-
Singleton	-	MH533020	ABPH49	*A. baumannii* AB49	China	149,960	50.8	243	-
Singleton	-	KX268652	vB_AbaS_TRS1	*A. baumannii* A118	UK/Argentina	40,749	40.26	70	[71]
Singleton	-	MH517022	SH-Ab 15599	*A. baumannii* 15599	China	143,204	38.46	177	-
Singleton	-	Unreleased	vB_AbaS_Arae	*A. baumannii* UKA9	UK	49,715	37.71	69	-

References

1. Jin, J.; Li, Z.J.; Wang, S.W.; Wang, S.M.; Huang, D.H.; Li, Y.H.; Ma, Y.Y.; Wang, J.; Liu, F.; Chen, X.D., *et al.* Isolation and characterization of zz1, a novel lytic phage that infects *acinetobacter baumannii* clinical isolates. *BMC Microbiol.* **2012**, *12*, 156. doi: 10.1186/1471-2180-12-156.

2. Jin, J.; Li, Z.J.; Wang, S.W.; Wang, S.M.; Chen, S.J.; Huang, D.H.; Zhang, G.; Li, Y.H.; Wang, X.T.; Wang, J.*, et al.* Genome organisation of the *acinetobacter* lytic phage zz1 and comparison with other t4-like *acinetobacter* phages. *BMC Genomics* **2014**, *15*, 793. doi: 10.1186/1471-2164-15-793.
3. Pulkkinen, E.; Wicklund, A.; Oduor, J.M.O.; Skurnik, M.; Kiljunen, S. Characterization of vb_apim_fhyaci03, a novel lytic bacteriophage that infects clinical *acinetobacter* strains. *Arch. Virol.* **2019**, *164*, 2197-2199. doi: 10.1007/s00705-019-04284-z.
4. Jansen, M.; Wahida, A.; Latz, S.; Krüttgen, A.; Häfner, H.; Buhl, E.M.; Ritter, K.; Horz, H.P. Enhanced antibacterial effect of the novel t4-like bacteriophage karl-1 in combination with antibiotics against multi-drug resistant *acinetobacter baumannii*. *Scientific Reports* **2018**, *8*, 14140. doi: 10.1038/s41598-018-32344-y.
5. Petrov, V.M.; Ratnayaka, S.; Nolan, J.M.; Miller, E.S.; Karam, J.D. Genomes of the t4-related bacteriophages as windows on microbial genome evolution. *Virology Journal* **2010**, *7*, 292. doi: 10.1186/1743-422x-7-292.
6. Ackermann, H.W.; Krisch, H.M. A catalogue of t4-type bacteriophages. *Arch. Virol.* **1997**, *142*, 2329-2345. doi: 10.1007/s007050050246.
7. Petrov, V.M.; Nolan, J.M.; Bertrand, C.; Levy, D.; Desplats, C.; Krisch, H.M.; Karam, J.D. Plasticity of the gene functions for DNA replication in the t4-like phages. *J. Mol. Biol.* **2006**, *361*, 46-68. doi: 10.1016/j.jmb.2006.05.071.
8. Kim, J.H.; Oh, C.; Choresca, C.H.; Shin, S.P.; Han, J.E.; Jun, J.W.; Heo, S.J.; Kang, D.H.; Park, S.C. Complete genome sequence of bacteriophage phiac-1 infecting *acinetobacter soli* strain kz-1. *J. Virol.* **2012**, *86*, 13131-13132. doi: 10.1128/jvi.02454-12.
9. Peng, F.; Mi, Z.; Huang, Y.; Yuan, X.; Niu, W.; Wang, Y.; Hua, Y.; Fan, H.; Bai, C.; Tong, Y. Characterization, sequencing and comparative genomic analysis of vb_abam-ime-ab2, a novel lytic bacteriophage that infects multidrug-resistant *acinetobacter baumannii* clinical isolates. *BMC Microbiol.* **2014**, *14*, 181. doi: 10.1186/1471-2180-14-181.
10. Yan, W.; Banerjee, P.; Liu, Y.; Mi, Z.; Bai, C.; Hu, H.; To, K.K.W.; Duong, H.T.T.; Leung, S.S.Y. Development of thermosensitive hydrogel wound dressing containing *acinetobacter baumannii* phage against wound infections. *Int. J. Pharm.* **2021**, *602*, 120508. doi: 10.1016/j.ijpharm.2021.120508.
11. Jeon, J.; Ryu, C.M.; Lee, J.Y.; Park, J.H.; Yong, D.; Lee, K. *In vivo* application of bacteriophage as a potential therapeutic agent to control oxa-66-like carbapenemase-producing *acinetobacter baumannii* strains belonging to sequence type 357. *Appl. Environ. Microbiol.* **2016**, *82*, 4200-4208. doi: 10.1128/aem.00526-16.
12. Jeon, J.; D'Souza, R.; Pinto, N.; Ryu, C.M.; Park, J.; Yong, D.; Lee, K. Characterization and complete genome sequence analysis of two myoviral bacteriophages infecting clinical carbapenem-resistant *acinetobacter baumannii* isolates. *J. Appl. Microbiol.* **2016**, *121*, 68-77. doi: 10.1111/jam.13134.
13. Guo, Z.; Huang, H.; Wu, X.; Hao, Y.; Sun, Y. Complete genome sequence of lytic bacteriophage lz35 infecting *acinetobacter baumannii* isolates. *Genome Announcements* **2016**, *4*. doi: 10.1128/genomeA.01104-16.
14. Wang, C.; Li, P.; Zhu, Y.; Huang, Y.; Gao, M.; Yuan, X.; Niu, W.; Liu, H.; Fan, H.; Qin, Y.*, et al.* Identification of a novel *acinetobacter baumannii* phage-derived depolymerase and its therapeutic application in mice. *Frontiers in Microbiology* **2020**, *11*, 1407. doi: 10.3389/fmicb.2020.01407.
15. Zhou, W.; Feng, Y.; Zong, Z. Two new lytic bacteriophages of the *myoviridae* family against carbapenem-resistant *acinetobacter baumannii*. *Frontiers in Microbiology* **2018**, *9*, 850. doi: 10.3389/fmicb.2018.00850.

16. Jiang, L.; Tan, J.; Hao, Y.; Wang, Q.; Yan, X.; Wang, D.; Tuo, L.; Wei, Z.; Huang, G. Isolation and characterization of a novel myophage abp9 against pandrug resistant *acinetobacater baumannii*. *Frontiers in Microbiology* **2020**, *11*, 506068. doi: 10.3389/fmicb.2020.506068.
17. Jiang, L.; Tan, J.; Hao, Y.; Wang, Q.; Yan, X.; Wang, D.; Tuo, L.; Wei, Z.; Huang, G. Corrigendum: Isolation and characterization of a novel myophage abp9 against pandrug resistant *acinetobacater baumannii*. *Frontiers in Microbiology* **2020**, *11*, 625283. doi: 10.3389/fmicb.2020.625283.
18. Popova, A.V.; Zhilenkov, E.L.; Myakinina, V.P.; Krasilnikova, V.M.; Volozhantsev, N.V. Isolation and characterization of wide host range lytic bacteriophage ap22 infecting *acinetobacter baumannii*. *FEMS Microbiol. Lett.* **2012**, *332*, 40-46. doi: 10.1111/j.1574-6968.2012.02573.x.
19. Dubrovin, E.V.; Popova, A.V.; Kraevskiy, S.V.; Ignatov, S.G.; Ignatyuk, T.E.; Yaminsky, I.V.; Volozhantsev, N.V. Atomic force microscopy analysis of the *acinetobacter baumannii* bacteriophage ap22 lytic cycle. *PLoS One* **2012**, *7*, e47348. doi: 10.1371/journal.pone.0047348.
20. Popova, A.V.; Miakinina, V.P.; Platonov, M.E.; Volozhantsev, N.V. [molecular characterization of the multidrug-resistant *acinetobacter baumannii* strains and assessment of their sensitivity to the phage ap22]. *Molecular Genetics, Microbiology, and Virusology* **2012**, 18-22. doi.
21. Yang, H.; Liang, L.; Lin, S.; Jia, S. Isolation and characterization of a virulent bacteriophage ab1 of *acinetobacter baumannii*. *BMC Microbiol.* **2010**, *10*, 131. doi: 10.1186/1471-2180-10-131.
22. Li, P.; Chen, B.; Song, Z.; Song, Y.; Yang, Y.; Ma, P.; Wang, H.; Ying, J.; Ren, P.; Yang, L.*, et al.* Bioinformatic analysis of the *acinetobacter baumannii* phage ab1 genome. *Gene* **2012**, *507*, 125-134. doi: 10.1016/j.gene.2012.07.029.
23. Merabishvili, M.; Vandenheuvel, D.; Kropinski, A.M.; Mast, J.; De Vos, D.; Verbeken, G.; Noben, J.P.; Lavigne, R.; Vaneechoutte, M.; Pirnay, J.P. Characterization of newly isolated lytic bacteriophages active against *acinetobacter baumannii*. *PLoS One* **2014**, *9*, e104853. doi: 10.1371/journal.pone.0104853.
24. Merabishvili, M.; Monserez, R.; van Belleghem, J.; Rose, T.; Jennes, S.; De Vos, D.; Verbeken, G.; Vaneechoutte, M.; Pirnay, J.P. Stability of bacteriophages in burn wound care products. *PLoS One* **2017**, *12*, e0182121. doi: 10.1371/journal.pone.0182121.
25. Essoh, C.; Vernadet, J.P.; Vergnaud, G.; Coulibaly, A.; Kakou-N'Douba, A.; N'Guetta, A.S.; Resch, G.; Pourcel, C. Complete genome sequences of five *acinetobacter baumannii* phages from abidjan, côte d'ivoire. *Microbiology Resource Announcements* **2019**, *8*. doi: 10.1128/mra.01358-18.
26. Asif, M.; Alvi, I.A.; Tabassum, R.; Rehman, S.U. Tac1, an unclassified bacteriophage of the family myoviridae infecting *acinetobacter baumannii* with a large burst size and a short latent period. *Arch. Virol.* **2020**, *165*, 419-424. doi: 10.1007/s00705-019-04483-8.
27. Wu, N.; Dai, J.; Guo, M.; Li, J.; Zhou, X.; Li, F.; Gao, Y.; Qu, H.; Lu, H.; Jin, J.*, et al.* Pre-optimized phage therapy on secondary *acinetobacter baumannii* infection in four critical covid-19 patients. *Emerging Microbes & Infections* **2021**, *10*, 612-618. doi: 10.1080/22221751.2021.1902754.
28. Jeon, J.; Park, J.H.; Yong, D. Efficacy of bacteriophage treatment against carbapenem-resistant *acinetobacter baumannii* in *galleria mellonella* larvae and a mouse model of acute pneumonia. *BMC Microbiol.* **2019**, *19*, 70. doi: 10.1186/s12866-019-1443-5.
29. Popova, A.V.; Shneider, M.M.; Myakinina, V.P.; Bannov, V.A.; Edelstein, M.V.; Rubalskii, E.O.; Aleshkin, A.V.; Fursova, N.K.; Volozhantsev, N.V. Characterization of myophage am24 infecting *acinetobacter baumannii* of the k9 capsular type. *Arch. Virol.* **2019**, *164*, 1493-1497. doi: 10.1007/s00705-019-04208-x.
30. Vorob'ev, A.M.; Anurova, M.N.; Aleshkin, A.V.; Gushchin, V.A.; Vasina, D.V.; Antonova, N.P.; Kiseleva, I.A.; Rubalskii, E.O.; Zul'karneev, E.R.; Laishevtsev, A.I.*, et al.* Determination of bactericidal activity spectrum of recombinant endolysins of ecd7, am24, ap22, si3, and st11 bacteriophages. *Bulletin of Experimental Biology and Medicine* **2021**, *170*, 636-639. doi: 10.1007/s10517-021-05122-6.

31. Timoshina, O.Y.; Shneider, M.M.; Evseev, P.V.; Shchurova, A.S.; Shelenkov, A.A.; Mikhaylova, Y.V.; Sokolova, O.S.; Kasimova, A.A.; Arbatsky, N.P.; Dmitrenok, A.S.*, et al.* Novel *acinetobacter baumannii* bacteriophage aristophanes encoding structural polysaccharide deacetylase. *Viruses* **2021**, *13*. doi: 10.3390/v13091688.
32. Chang, K.C.; Lin, N.T.; Hu, A.; Lin, Y.S.; Chen, L.K.; Lai, M.J. Genomic analysis of bacteriophage φab1, a φkmv-like virus infecting multidrug-resistant *acinetobacter baumannii*. *Genomics* **2011**, *97*, 249-255. doi: 10.1016/j.ygeno.2011.01.002.
33. Huang, G.; Le, S.; Peng, Y.; Zhao, Y.; Yin, S.; Zhang, L.; Yao, X.; Tan, Y.; Li, M.; Hu, F. Characterization and genome sequencing of phage abp1, a new phikmv-like virus infecting multidrug-resistant *acinetobacter baumannii*. *Curr. Microbiol.* **2013**, *66*, 535-543. doi: 10.1007/s00284-013-0308-7.
34. Huang, G.; Shen, X.; Gong, Y.; Dong, Z.; Zhao, X.; Shen, W.; Wang, J.; Hu, F.; Peng, Y. Antibacterial properties of *acinetobacter baumannii* phage abp1 endolysin (plyab1). *BMC Infect. Dis.* **2014**, *14*, 681. doi: 10.1186/s12879-014-0681-2.
35. Yin, S.; Huang, G.; Zhang, Y.; Jiang, B.; Yang, Z.; Dong, Z.; You, B.; Yuan, Z.; Hu, F.; Zhao, Y.*, et al.* Phage abp1 rescues human cells and mice from infection by pan-drug resistant *acinetobacter baumannii*. *Cellular Physiology and Biochemistry* **2017**, *44*, 2337-2345. doi: 10.1159/000486117.
36. Zhang, J.; Liu, X.; Li, X.J. Bioinformatic analysis of phage ab3, a phikmv-like virus infecting *acinetobacter baumannii*. *Gen. Mol. Res.* **2015**, *14*, 190-198. doi: 10.4238/2015.January.16.2.
37. Kropinski, A.M. Accurate description of phages and their genomes--genet. Mol. Res. 14 (1): 190-198 "bioinformatic analysis of phage ab3, a phikmv-like virus infecting *acinetobacter baumannii*". *Gen. Mol. Res.* **2015**, *14*, 15902-15903. doi: 10.4238/2015.December.3.2.
38. Knirel, Y.A.; Shneider, M.M.; Popova, A.V.; Kasimova, A.A.; Senchenkova, S.N.; Shashkov, A.S.; Chizhov, A.O. Mechanisms of *acinetobacter baumannii* capsular polysaccharide cleavage by phage depolymerases. *Biochemistry (Moscow)* **2020**, *85*, 567-574. doi: 10.1134/s0006297920050053.
39. Lai, M.J.; Chang, K.C.; Huang, S.W.; Luo, C.H.; Chiou, P.Y.; Wu, C.C.; Lin, N.T. The tail associated protein of *acinetobacter baumannii* phage ϕab6 is the host specificity determinant possessing exopolysaccharide depolymerase activity. *PLoS One* **2016**, *11*, e0153361. doi: 10.1371/journal.pone.0153361.
40. Lee, I.M.; Yang, F.L.; Chen, T.L.; Liao, K.S.; Ren, C.T.; Lin, N.T.; Chang, Y.P.; Wu, C.Y.; Wu, S.H. Pseudaminic acid on exopolysaccharide of *acinetobacter baumannii* plays a critical role in phage-assisted preparation of glycoconjugate vaccine with high antigenicity. *J. Am. Chem. Soc.* **2018**, *140*, 8639-8643. doi: 10.1021/jacs.8b04078.
41. Shahed-Al-Mahmud, M.; Roy, R.; Sugiokto, F.G.; Islam, M.N.; Lin, M.D.; Lin, L.C.; Lin, N.T. Phage ϕab6-borne depolymerase combats *acinetobacter baumannii* biofilm formation and infection. *Antibiotics (Basel)* **2021**, *10*. doi: 10.3390/antibiotics10030279.
42. Wu, M.; Hu, K.; Xie, Y.; Liu, Y.; Mu, D.; Guo, H.; Zhang, Z.; Zhang, Y.; Chang, D.; Shi, Y. Corrigendum: A novel phage pd-6a3, and its endolysin ply6a3, with extended lytic activity against *acinetobacter baumannii*. *Frontiers in Microbiology* **2019**, *10*, 196. doi: 10.3389/fmicb.2019.00196.
43. Liu, Y.; Mi, Z.; Mi, L.; Huang, Y.; Li, P.; Liu, H.; Yuan, X.; Niu, W.; Jiang, N.; Bai, C.*, et al.* Identification and characterization of capsule depolymerase dpo48 from acinetobacter baumannii phage ime200. *PeerJ* **2019**, *7*, e6173. doi: 10.7717/peerj.6173.
44. Hua, Y.; Luo, T.; Yang, Y.; Dong, D.; Wang, R.; Wang, Y.; Xu, M.; Guo, X.; Hu, F.; He, P. Phage therapy as a promising new treatment for lung infection caused by carbapenem-resistant *acinetobacter baumannii* in mice. *Frontiers in Microbiology* **2017**, *8*, 2659. doi: 10.3389/fmicb.2017.02659.

45. Popova, A.V.; Lavysh, D.G.; Klimuk, E.I.; Edelstein, M.V.; Bogun, A.G.; Shneider, M.M.; Goncharov, A.E.; Leonov, S.V.; Severinov, K.V. Novel fri1-like viruses infecting *acinetobacter baumannii*-vb_abap_as11 and vb_abap_as12-characterization, comparative genomic analysis, and host-recognition strategy. *Viruses* **2017**, *9*. doi: 10.3390/v9070188.
46. Oliveira, H.; Costa, A.R.; Konstantinides, N.; Ferreira, A.; Akturk, E.; Sillankorva, S.; Nemec, A.; Shneider, M.; Dötsch, A.; Azeredo, J. Ability of phages to infect *acinetobacter calcoaceticus-acinetobacter baumannii* complex species through acquisition of different pectate lyase depolymerase domains. *Environ. Microbiol.* **2017**, *19*, 5060-5077. doi: 10.1111/1462-2920.13970.
47. Yuan, Y.; Wang, L.; Li, X.; Tan, D.; Cong, C.; Xu, Y. Efficacy of a phage cocktail in controlling phage resistance development in multidrug resistant *acinetobacter baumannii*. *Virus Res.* **2019**, *272*, 197734. doi: 10.1016/j.virusres.2019.197734.
48. Yuan, Y.; Li, X.; Wang, L.; Li, G.; Cong, C.; Li, R.; Cui, H.; Murtaza, B.; Xu, Y. The endolysin of the *acinetobacter baumannii* phage vb_abap_d2 shows broad antibacterial activity. *Microbial Biotechnology* **2021**, *14*, 403-418. doi: 10.1111/1751-7915.13594.
49. Popova, A.V.; Shneider, M.M.; Arbatsky, N.P.; Kasimova, A.A.; Senchenkova, S.N.; Shashkov, A.S.; Dmitrenok, A.S.; Chizhov, A.O.; Mikhailova, Y.V.; Shagin, D.A.*, et al.* Specific interaction of novel friunavirus phages encoding tailspike depolymerases with corresponding *acinetobacter baumannii* capsular types. *J. Virol.* **2020**, *95*. doi: 10.1128/jvi.01714-20.
50. Abdelkader, K.; Gutiérrez, D.; Grimon, D.; Ruas-Madiedo, P.; Lood, C.; Lavigne, R.; Safaan, A.; Khairalla, A.S.; Gaber, Y.; Dishisha, T.*, et al.* Lysin lysmk34 of *acinetobacter baumannii* bacteriophage pmk34 has a turgor pressure-dependent intrinsic antibacterial activity and reverts colistin resistance. *Appl. Environ. Microbiol.* **2020**, *86*. doi: 10.1128/aem.01311-20.
51. Grygorcewicz, B.; Roszak, M.; Golec, P.; Śleboda-Taront, D.; Łubowska, N.; Górska, M.; Jursa-Kulesza, J.; Rakoczy, R.; Wojciuk, B.; Dołęgowska, B. Antibiotics act with vb_abap_agc01 phage against *acinetobacter baumannii* in human heat-inactivated plasma blood and galleria mellonella models. *International Journal of Molecular Sciences* **2020**, *21*. doi: 10.3390/ijms21124390.
52. Hernandez-Morales, A.C.; Lessor, L.L.; Wood, T.L.; Migl, D.; Mijalis, E.M.; Cahill, J.; Russell, W.K.; Young, R.F.; Gill, J.J.; Pfeiffer, J.K. Genomic and biochemical characterization of acinetobacter podophage petty reveals a novel lysis mechanism and tail-associated depolymerase activity. *J. Virol.* **2018**, *92*, e01064-01017. doi: doi:10.1128/JVI.01064-17.
53. Mumm, I.P.; Wood, T.L.; Chamakura, K.R.; Everett, G.F.K. Complete genome of acinetobacter baumannii podophage petty. *Genome Announcements* **2013**, *1*, e00850-00813. doi: doi:10.1128/genomeA.00850-13.
54. Mendes, J.J.; Leandro, C.; Mottola, C.; Barbosa, R.; Silva, F.A.; Oliveira, M.; Vilela, C.L.; Melo-Cristino, J.; Górski, A.; Pimentel, M.*, et al. In vitro* design of a novel lytic bacteriophage cocktail with therapeutic potential against organisms causing diabetic foot infections. *J. Med. Microbiol.* **2014**, *63*, 1055-1065. doi: 10.1099/jmm.0.071753-0.
55. López-Leal, G.; Reyes-Muñoz, A.; Santamaria, R.I.; Cevallos, M.A.; Pérez-Monter, C.; Castillo-Ramírez, S. A novel vieuvirus from multidrug-resistant *acinetobacter baumannii*. *Arch. Virol.* **2021**, *166*, 1401-1408. doi: 10.1007/s00705-021-05010-4.
56. Jeon, J.; Kim, J.-w.; Yong, D.; Lee, K.; Chong, Y. Complete genome sequence of the podoviral bacteriophage ymc/09/02/b1251 aba bp, which causes the lysis of an oxa-23-producing carbapenem-resistant acinetobacter baumannii isolate from a septic patient. *J. Virol.* **2012**, *86*, 12437-12438. doi: doi:10.1128/JVI.02132-12.

57. Jeon, J.; D'Souza, R.; Pinto, N.; Ryu, C.M.; Park, J.H.; Yong, D.; Lee, K. Complete genome sequence of the siphoviral bacteriophage βφ-r3177, which lyses an oxa-66-producing carbapenem-resistant *acinetobacter baumannii* isolate. *Arch. Virol.* **2015**, *160*, 3157-3160. doi: 10.1007/s00705-015-2604-y.
58. Blasco, L.; Ambroa, A.; Lopez, M.; Fernandez-Garcia, L.; Bleriot, I.; Trastoy, R.; Ramos-Vivas, J.; Coenye, T.; Fernandez-Cuenca, F.; Vila, J.*, et al.* Combined use of the ab105-2ϕδci lytic mutant phage and different antibiotics in clinical isolates of multi-resistant *acinetobacter baumannii*. *Microorganisms* **2019**, *7*. doi: 10.3390/microorganisms7110556.
59. Turner, D.; Wand, M.E.; Briers, Y.; Lavigne, R.; Sutton, J.M.; Reynolds, D.M. Characterisation and genome sequence of the lytic *acinetobacter baumannii* bacteriophage vb_abas_loki. *PLoS One* **2017**, *12*, e0172303. doi: 10.1371/journal.pone.0172303.
60. Farmer, N.G.; Wood, T.L.; Chamakura, K.R.; Kuty Everett, G.F. Complete genome of *acinetobacter baumannii* n4-like podophage presley. *Genome Announcements* **2013**, *1*. doi: 10.1128/genomeA.00852-13.
61. Cheng, M.; Luo, M.; Xi, H.; Zhao, Y.; Le, S.; Chen, L.K.; Tan, D.; Guan, Y.; Wang, T.; Han, W.*, et al.* The characteristics and genome analysis of vb_apip_xc38, a novel phage infecting *acinetobacter pittii*. *Virus Genes* **2020**, *56*, 498-507. doi: 10.1007/s11262-020-01766-0.
62. Xu, J.; Li, X.; Kang, G.; Bai, L.; Wang, P.; Huang, H. Isolation and characterization of abtj, an *acinetobacter baumannii* phage, and functional identification of its receptor-binding modules. *Viruses* **2020**, *12*. doi: 10.3390/v12020205.
63. Hua, Y.; Xu, M.; Wang, R.; Zhang, Y.; Zhu, Z.; Guo, M.; He, P. Characterization and whole genome analysis of a novel bacteriophage sh-ab 15497 against multidrug resistant *acinetobacater baummanii*. *Acta Biochimica et Biophysica Sinica* **2019**, *51*, 1079-1081. doi: 10.1093/abbs/gmz094.
64. Pehde, B.M.; Niewohner, D.; Keomanivong, F.E.; Carruthers, M.D. Genome sequence and characterization of *acinetobacter* phage dmu1. *PHAGE* **2021**, *2*, 50-56. doi: 10.1089/phage.2020.0043.
65. Badawy, S.; Pajunen, M.I.; Haiko, J.; Baka, Z.A.M.; Abou-Dobara, M.I.; El-Sayed, A.K.A.; Skurnik, M. Identification and functional analysis of temperate siphoviridae bacteriophages of *acinetobacter baumannii*. *Viruses* **2020**, *12*. doi: 10.3390/v12060604.
66. Oliveira, H.; Costa, A.R.; Ferreira, A.; Konstantinides, N.; Santos, S.B.; Boon, M.; Noben, J.P.; Lavigne, R.; Azeredo, J. Functional analysis and antivirulence properties of a new depolymerase from a myovirus that infects *acinetobacter baumannii* capsule k45. *J. Virol.* **2019**, *93*. doi: 10.1128/jvi.01163-18.
67. Soothill, J.S. Treatment of experimental infections of mice with bacteriophages. *J. Med. Microbiol.* **1992**, *37*, 258-261. doi: 10.1099/00222615-37-4-258.
68. Henein, A.E.; Hanlon, G.W.; Cooper, C.J.; Denyer, S.P.; Maillard, J.Y. A partially purified *acinetobacter baumannii* phage preparation exhibits no cytotoxicity in 3t3 mouse fibroblast cells. *Frontiers in Microbiology* **2016**, *7*, 1198. doi: 10.3389/fmicb.2016.01198.
69. Popova, A.V.; Shneider, M.M.; Mikhailova, Y.V.; Shelenkov, A.A.; Shagin, D.A.; Edelstein, M.V.; Kozlov, R.S. Complete genome sequence of *acinetobacter baumannii* phage bs46. *Microbiology Resource Announcements* **2020**, *9*. doi: 10.1128/mra.00398-20.
70. Buttimer, C.; O'Sullivan, L.; Elbreki, M.; Neve, H.; McAuliffe, O.; Ross, R.P.; Hill, C.; O'Mahony, J.; Coffey, A. Genome sequence of jumbo phage vb_abam_me3 of *acinetobacter baumanni*. *Genome Announcements* **2016**, *4*. doi: 10.1128/genomeA.00431-16.
71. Turner, D.; Wand, M.E.; Sutton, J.M.; Centron, D.; Kropinski, A.M.; Reynolds, D.M. Genome sequence of vb_abas_trs1, a viable prophage isolated from *acinetobacter baumannii* strain a118. *Genome Announcements* **2016**, *4*. doi: 10.1128/genomeA.01051-16.
